# Novel CD44-targeting and pH/redox-dual-stimuli-responsive core–shell nanoparticles loading triptolide combats breast cancer growth and lung metastasis

**DOI:** 10.1186/s12951-021-00934-0

**Published:** 2021-06-23

**Authors:** Jinfeng Shi, Yali Ren, Jiaqi Ma, Xi Luo, Jiaxin Li, Yihan Wu, Huan Gu, Chaomei Fu, Zhixing Cao, Jinming Zhang

**Affiliations:** State Key Laboratory of Southwestern Chinese Medicine Resources, Pharmacy School, College of Pharmacy, Chengdu University of Traditional Chinese Medicine, No. 1166 Liutai Avenue, Wenjiang District, Chengdu, China

**Keywords:** Triptolide, Nanoparticles, Breast cancer, Lung metastasis, Low toxicity

## Abstract

**Background:**

The toxicity and inefficient delivery of triptolide (TPL) in tumor therapy have greatly limited the clinical application. Thus, we fabricated a CD44-targeting and tumor microenvironment pH/redox-sensitive nanosystem composed of hyaluronic acid-vitamin E succinate and poly (β-amino esters) (PBAEss) polymers to enhance the TPL-mediated suppression of breast cancer proliferation and lung metastasis.

**Results:**

The generated TPL nanoparticles (NPs) had high drug loading efficiency (94.93% ± 2.1%) and a desirable average size (191 nm). Mediated by the PBAEss core, TPL/NPs displayed a pH/redox-dual-stimuli-responsive drug release profile in vitro. Based on the hyaluronic acid coating, TPL/NPs exhibited selective tumor cellular uptake and high tumor tissue accumulation capacity by targeting CD44. Consequently, TPL/NPs induced higher suppression of cell proliferation, blockage of proapoptotic and cell cycle activities, and strong inhibition of cell migration and invasion than that induced by free TPL in MCF-7 and MDA-MB-231 cells. Importantly, TPL/NPs also showed higher efficacy in shrinking tumor size and blocking lung metastasis with decreased systemic toxicity in a 4T1 breast cancer mouse model at an equivalent or lower TPL dosage compared with that of free TPL. Histological immunofluorescence and immunohistochemical analyses in tumor and lung tissue revealed that TPL/NPs induced a high level of apoptosis and suppressed expression of matrix metalloproteinases, which contributed to inhibiting tumor growth and pulmonary metastasis.

**Conclusion:**

Collectively, our results demonstrate that TPL/NPs, which combine tumor active targeting and pH/redox-responsive drug release with proapoptotic and antimobility effects, represent a promising candidate in halting breast cancer progression and metastasis while minimizing systemic toxicity.

**Graphic Abstract:**

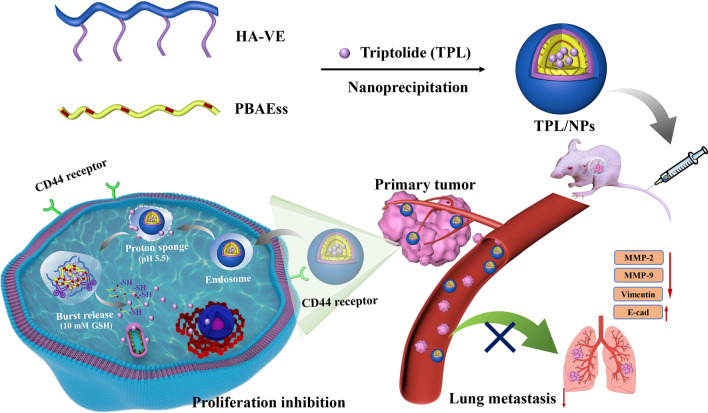

**Supplementary Information:**

The online version contains supplementary material available at 10.1186/s12951-021-00934-0.

## Background

Globally, breast cancer is still one of the highest causes of morbidity from malignant tumors and remains the most common lethal cancer found in females [1, 2]. Rapid tumor growth and fast metastasis are the predominant causes of death in patients with breast cancer, with a low median 5 years survival rate of approximately 26% [3]. Despite several clinical strategies, including surgery, radiotherapy, and chemotherapy, being currently used to eliminate tumors and inhibit their metastatic spread, the treatment efficacy remains limited, and the overall survival may only be improved for a few months at most [4]. Particularly, the poor therapeutic outcome of chemotherapy, as the most mainstream treatment approach, is attributed to insufficient delivery efficiency to the tumor site and/or the antimetastatic ability of the anticancer agents.

Triptolide (TPL) is a natural compound isolated from a Chinese medicinal herb *Tripterygium wilfordii* Hook F. TPL has various biological activities and has attracted extensive attention in the medicinal field. Particularly, TPL processes substantial anticancer effects against a wide range of cancers [5, 6] with several signaling pathways involved, including those involved in cell apoptosis and in inhibition of cell proliferation and suppression of tumor metastasis [7]. However, despite these anticancer benefits, several challenges remain, including the well-acknowledged high toxicity [8], poor solubility, and limited drug delivery efficiency to tumor sites that have severely impeded the potential clinical value of TPL. Recently, several strategies have been utilized to develop TPL-targeted delivery systems [9], including direct conjugation of TPL to tumor targeting ligands such as sugar, short peptide, oligonucleotide, and antibody [10, 11]. However, the polymer-/ligand-drug conjugates are apt to confront the rapid clearance or premature drug release in the blood circulation.

In comparison with the instability of drug-ligand conjugation during transportation in the bloodstream, drugs loaded in nanosystems exhibit higher stability, long-circulating capacity, and higher accumulation efficiency at the tumor site by the enhanced permeability and retention (EPR) effect. Multiple studies have shown that the interaction of hyaluronic acid (HA) and the CD44 receptor play an essential role in cancer cell proliferation and migration, inflammation, and tumor growth [12]. Many kinds of HA-modified/coating nanocarriers have been investigated to target CD44-overexpressing cancer cells to take advantage of this interaction [13]. Moreover, these encapsulated drugs in nanoparticles with a stable core–shell structure must produce a drug burst at the cancer site, which is selectively triggered by internal or external stimuli, and simultaneously avoid drug leakage during circulation in the blood [14]. The unique pathophysiology of tumors exhibits acidic pH values in both tumor tissue (~ pH 6.5) and late endosomes/lysosomes (< pH 5.5) [15, 16]. Poly (β-amino ester) (PBAE) is a cationic-charged biodegradable polymer that has been widely applied as a biodegradable and biocompatible material for anticancer drug delivery. Additionally, the rapid protonation of amino groups in PBAE frameworks upon exposure to acidic pH can induce “proton sponge” effects of PBAE-based nanoparticles that facilitate the escape of encapsulated drugs from lysosomes [17, 18]. Additionally, redox-responsive nanoparticles have also aroused considerable enthusiasm because of the high intracellular concentration of glutathione (GSH) (approximately 2–10 mM) in tumors in comparison with that in the extracellular environment (2–20 μM) [19]. Several pH- [20] or redox-responsive [21, 22] nanoparticles loading TPL have been reported to benefit the delivery of TPL with enhanced antitumor effects and reduced toxicity to normal tissues. Nonetheless, although these functional nano-vehicles including tumor-targeting/ pH-sensitive/ redox-responsive NPs were used for the encapsulation of TPL, few studies were implemented to combine the advantages of tumor-targeting and tumor microenvironment responsive drug release. The nanocarriers with dual- or multiple-stimuli-responsive characters would exhibit more sensitive and complete drug release than those with a single sensitive mechanism.

As a proof of concept, we designed a novel multifunctional nanosized system with the tumor active targeting and pH/redox-dual-stimuli-responsive properties to encapsulate TPL for enhanced treatment of breast cancer growth and metastasis. Specifically, a novel disulfide-containing poly(β-amino ester) (PBAEss) copolymer was synthesized in response to both pH and redox dual triggers and acted as the hydrophobic core for loading TPL. Then, vitamin E succinate (VE) as a hydrophobic side chain was conjugated with HA to generate an amphiphilic copolymer (HA-VE), which would produce a shell coating on the PBAEss nanocores. Hence, TPL loaded in this multifunctional nanosystem (TPL/NPs) composed of HA-VE and PBAEss copolymers would be accumulated at the tumor site through the EPR effect and then via HA-CD44 binding with CD44 receptors be internalized into CD44 overexpressing breast cancer cells [13]. Subsequently, both the acidic pH of endosomes and high GSH in cytoplasm will accelerate the rapid escape of TPL from HA-VE/PBAEss NPs mediated by the “proton sponge” effect and disulfide bond cleavage of the PBAEss nanocore. Ultimately, this rapid accumulation of TPL at the tumor site would be beneficial to inducing cancer cell apoptosis and suppressing lung metastasis.

## Results

### Copolymer synthesis and characterization

**Fig. 1:**
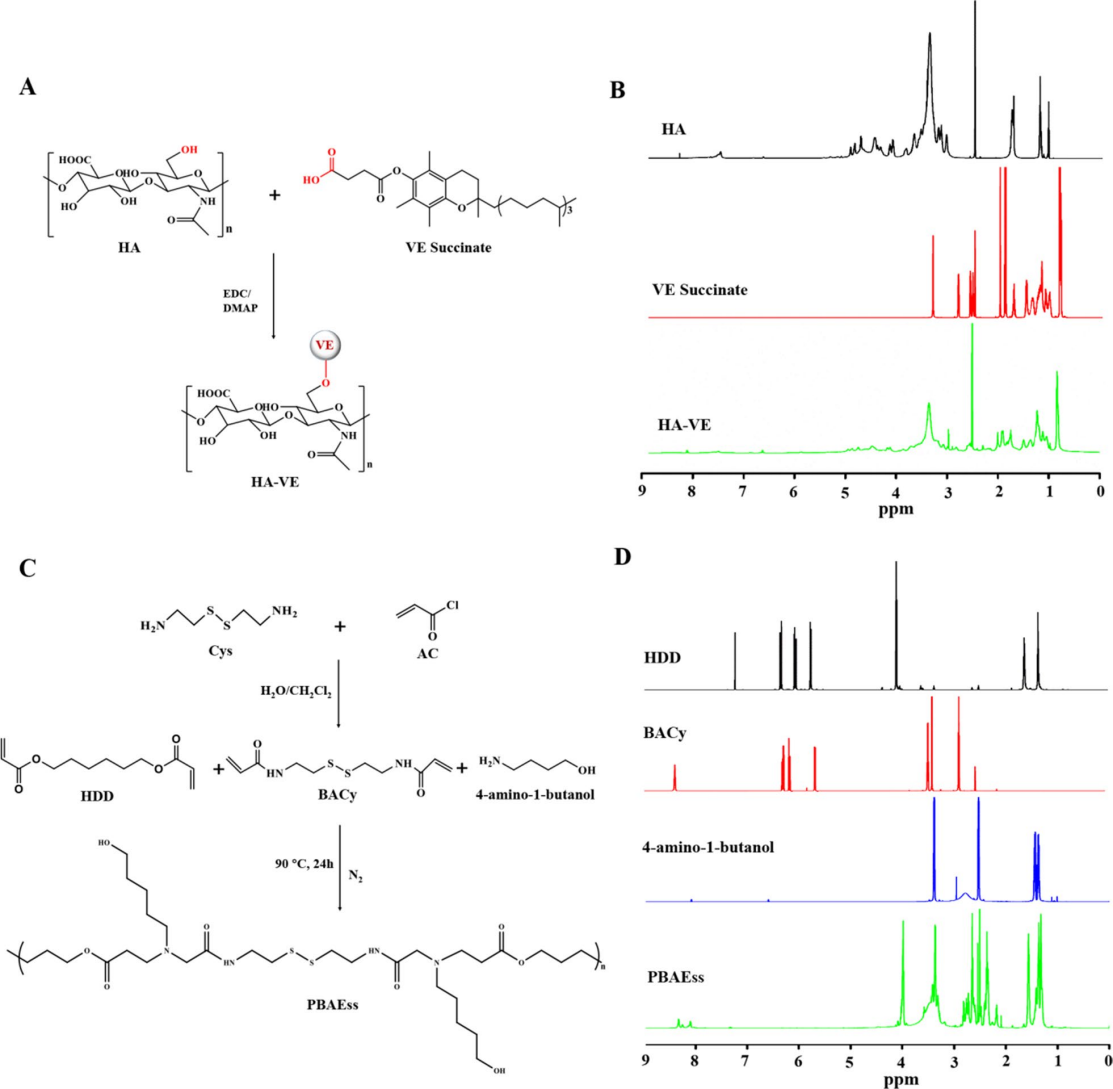
**a** Synthesis route of HA-VE conjugate. **b** ^1^H NMR spectra of HA, VE, and HA-VE in DMSO-d_6_. **c** Synthesis route of PBAEss copolymer. **d** ^1^H NMR spectra of HDD, BACy, 4-amino-1-butanol, and PBAEss in CDCl_3_

VE was conveniently conjugated to HA with an esterification reaction in one synthesis step in the presence of 1-(3-dimethylaminopropyl)-3-ethylcarbodiimide hydrochloride (EDC) and 4-(dimethylamino)-pyridin (DMAP), as demonstrated in Fig. 1a. The synthesis of HA-VE conjugate was confirmed by ^1^H nuclear magnetic resonance (NMR) analysis. As shown in Fig. 1b, the characteristic peaks for methylene groups at the sugar unit and *N*-acetyl group of HA were identified at 3.10–4.00 and approximately 1.80 ppm [23]. Intense peaks appeared at 0.80 and 1.00–2.00 ppm, which represented the methyl group and methylene on the fatty acid chain of VE, respectively [24]. These characteristic peaks could be observed in the ^1^H NMR of HA-VE conjugate, indicating that this had been successfully synthesized.

PBAEss were synthesized with a yield of 72.3% as mediated by the Michael addition reaction, as shown in Fig. 1c. The molecular weight of the PBAEss copolymer was determined by gel permeation chromatography (GPC) analysis (Additional file 1: Fig. S1). The molecular weight (Mw) and Mn of PBAEss were 3.51 × 10^4^ and 1.75 × 10^4^, respectively (Mw/Mn = 2.00). The chemical structures of these intermediates, including 4-amino-1-butanol, 1,6-hexanediol diacrylate (HDD), and N, N′-bis(acryloyl)cystamine (BACy), and the synthesized PBAEss copolymer were characterized by ^1^H NMR spectra (Fig. 1d). As shown, the characteristic peaks at 4.2 and 5.5–6.5 ppm in the HDD spectrum were attributed to–OCH_2_–and vinyl protons. In the BACy spectrum, the peaks near 3.40 and 5.5–6.5 ppm were assigned to the proton shifts of–SCH_2_–and the characteristic protons of vinyl protons, respectively. However, after polymerization between the vinyl groups of HDD and BACy and the amine group of 4-amino-1-butanol, the chemical shift derived from the vinyl protons disappeared in the PBAEss spectrum. Meanwhile, the characteristic methylene proton peaks of NH_2_CH_2_–, –CH_2_–CH_2_–, and –CH_2_OH in 4-amino-1-butanol that appeared at 2.46, 1.2–1.5, and 3.4 ppm, respectively, also could be found in the ^1^H NMR spectrum of PBAEss. Altogether, the above results indicated that PBAEss was successfully synthesized.

### Preparation and characterization of TPL/NPs

PBAEss analogs have been previously demonstrated to form NPs for efficient encapsulation of genes or anticancer drugs by self-assembly on the basis of their amphiphilic properties. Here we encapsulated TPL into the PBAEss nanocore using the nanoprecipitation method. Meanwhile, the hydrophilic HA-VE shell was coated on PBAEss nanocore by electrical charge interaction. To obtain NPs with high drug loading efficiency and suitable size distribution, different compositions of HA-VE and PBAEss copolymer were used (Additional file 1: Table S1), including 10, 10, and 2 mg HA-VE, PBAEss, and TPL, respectively. The collected TPL loaded in HA-VE/PBAEss NPs (TPL/NPs) had an average diameter of approximately 191 nm with a relatively narrow distribution (Table 1). The drug loading efficiency and drug entrapment efficiency of TPL loading in NPs were determined as 8.63% ± 0.72% and 94.93% ± 2.1%, respectively.

**Table 1:** Characterization of TPL/NPs

Sample	Particle size (nm)	PDI^a^	Zeta potential (mV)	DLE^b^ (%)	DEE^c^ (%)
Blank NPs	184.53 ± 7.52	0.195 ± 0.052	− 6.33 ± 0.32	–	–
TPL/NPs	191.31 ± 7.08	0.176 ± 0.040	− 6.94 ± 0.58	8.63 ± 0.72	94.93 ± 2.1

^a^Polydispersity index

^b^Drug loading efficiency

^c^Drug entrapment efficiency

**Fig. 2:**
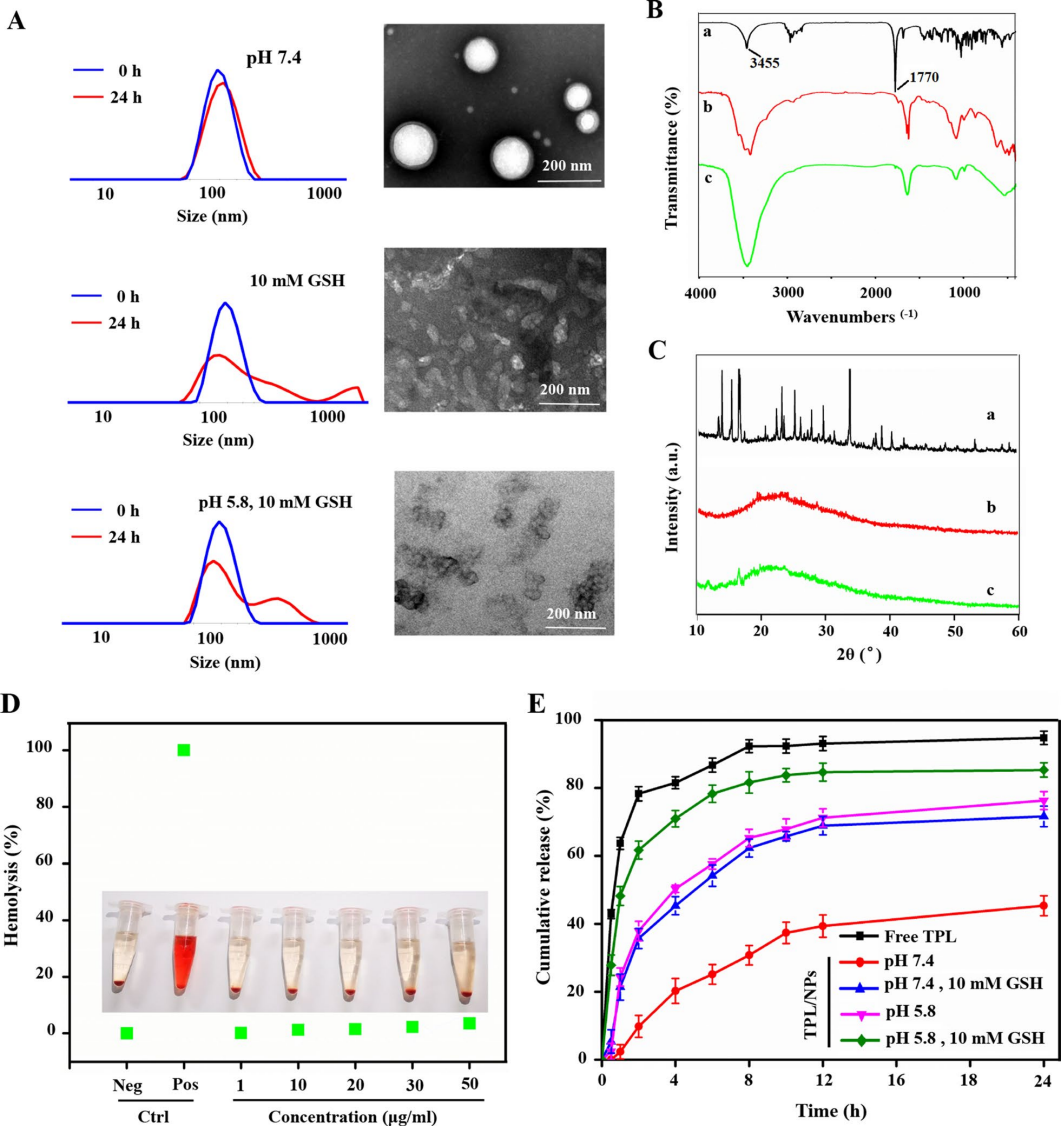
Characterization of TPL/NPs. **a** Particle distribution by DLS and TEM image of TPL/NPs in response to different conditions. FTIR (**b**) and XRD (**c**) analysis of TPL/NPs. (**a**: Free TPL; **b**: TPL + polymer; c: TPL/NPs). **d** Hemolysis assay of TPL/NPs at different concentrations of HA-VE/PBAEss polymers; PBS and water were employed as negative and positive controls, respectively. **e** In vitro release profile of TPL from TPL/NPs in different mediums at 37 ℃

The size distribution of TPL/NPs via dynamic light scattering (DLS) and morphology via transmission electron microscopy (TEM) observation are shown in Fig. 2A. TPL/NPs had a uniform size distribution, regularly spherical shape, and compact structure. However, in response to either reductive (GSH 10 mM) or acid condition (pH 5.8) to mimic the redox and acid tumor microenvironment, the size distribution of TPL/NPs began to increase with multiple peaks. The uniform orbicular morphology of TPL/NPs dispersed and fragmented. Additional file 1: Fig. S2 shown the positive and negative zeta potential of PBAEss and HA-VE solution, respectively. Additional file 1: Fig. S3 shown the size changes of TPL/NPs in response to different conditions. The above results demonstrate that the TPL/NPs with high drug loading and narrow particle size possessed the pH- and redox-dual-stimuli-responsive properties that were suitable for the controlled release profiles in the tumor microenvironment. Moreover, as depicted in Additional file 1: Fig. S4, TPL/NPs remained stable at a particle size below 200 nm in either pH 7.4 phosphate-buffered saline (PBS) or in 10% serum-supplemented Dulbecco’s modified Eagle medium (DMEM) at 4 °C for 96 h in situ. After continuous storage for 14 days, TPL/NPs exhibited a stable size distribution, with relative standard deviation (RSD) values of an average size of 3.3% (Additional file 1: Fig. S5). These results indicated the favorable stability of TPL/NPs, attributed to their uniform size distribution and anionic-based electrostatic repulsion.

Fourier transform infrared spectroscopy (FTIR) and X-ray diffraction (XRD) were applied to evaluate the intermolecular interactions between TPL and HA-VE/PBAEss nanovehicles. In Fig. 2B, the strong characteristic peaks at 3455 and 1770 cm^−1^ are shown in the FTIR spectrum of TPL and were derived from (a) the hydroxyl group and the carbonyl group in the lactonic ring, respectively, but were absent in the FTIR spectrum of blank NPs (b); the disappearance of these peaks in TPL/NPs (c) on the same chemical shift suggested that TPL was totally encapsulated in polymeric NPs, instead of in a physical mixture. Additionally, in the XRD spectrum (Fig. 2C), because of the crystal structure of TPL, the characteristic crystallization peaks mainly arranged between 10° and 40°. Thus, after loading in NPs, TPL/NPs transformed into an amorphous form, indicating that there was no crystalline material in the TPL/NPs.

To ensure the biosafety of TPL/NPs for intravenous injection, hemolysis analysis was also conducted. We examined the effect of TPL/NPs on the hemolytic profile of red blood cells; PBS and water were used as the negative and positive control, respectively. Expectedly, the TPL/NPs at 1–50 µg/mL did not induce erythrocytes to release hemoglobin (hemolysis rate of ≤ 5%) (Fig. 2D). This indicated the safety of TPL/NPs and their compatibility for intravenous administration.

### In vitro drug release of TPL from TPL/NPs

In view of the pH/redox-sensitive properties demonstrated by size changes above, the in vitro TPL release profiles from TPL/NPs in different mediums were measured using the dialysis method and were assessed at the physiological condition (pH 7.4), redox condition only (pH 7.4, 10 mM GSH), acid condition only (pH 5.8), and simulated tumor intracellular condition (pH 5.8, 10 mM GSH). Free TPL could be rapidly released as a control, whereas the cumulative release of TPL reached approximately 90% of free TPL over 8 h (Fig. 2E). At pH 7.4, TPL was gently released from NPs, and only 45% of TPL was released from NPs over 24 h, which indicated that the TPL molecules were well protected in the core of HA-VE/PBAEss NPs. There was much faster drug release from TPL/NPs in response to either 10 mM GSH or pH 5.8 buffer compared with that in pH 7.4 buffer. In response to 10 mM GSH, the PBAEss copolymer was expected to fracture because of the cleavage of disulfide bonds. When the pH was decreased to 5.8, the drug release rate sharply accelerated because of the protonation of tertiary amine residues in the PBAEss segment. Thus, the cumulative release of TPL for 24 h was 71.65% (10 mM GSH buffer) and 76.31% (pH 5.8 buffer), respectively, indicating the intense initial burst release. The fastest drug release profile was at pH 5.8 with 10 mM GSH because of the combination of disulfide bond cleavage and tertiary amine protonation. The cumulative release of TPL reached 85.32% over 24 h. Taken together, TPL loaded in HA-VE/PBAEss NPs efficiently undergo controlled release in physiological conditions and a selectively rapid drug release in the tumor microenvironment.

### TPL/NPs cytotoxicity against breast cancer cells

**Fig. 3:**
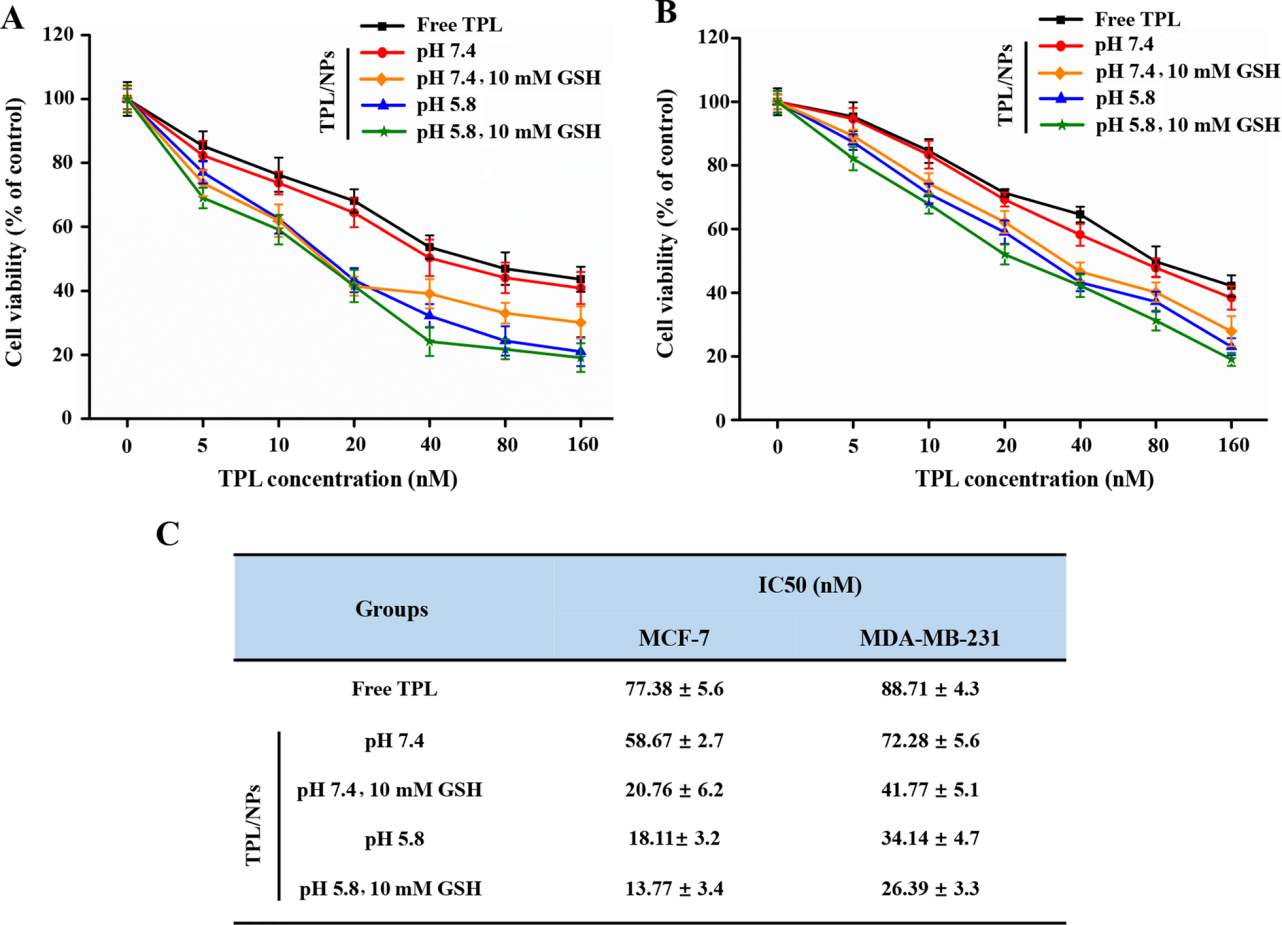
Cytotoxicity of TPL/NPs against MDA-MB-231 (**a**) and MCF-7 (**b**) cells with 48 h treatment in different culture media. **c** IC_50_ values of TPL/NPs in MDA-MB-231 and MCF-7 cells after 48 h treatment

We investigated the in vitro cytotoxicity of TPL/NPs in different media (i.e., pH 7.4, pH 7.4 + 10 mM GSH, pH 5.8, and pH 5.8 + 10 mM GSH) against MDA-MB-231 and MCF-7 cells after 48 h incubation with 3-(4,5-dimethylthiazol-2-yl)-2,5-diphenyltetrazoliumbromide (MTT). A pH of 5.8 and a GSH concentration of 10 mM in the culture medium were chosen to represent the early endosome and cytoplasm of cancer cells. All the TPL formulas suppressed proliferation of MDA-MB-231 and MCF-7 cells in a dose-dependent manner from 0 to 160 nM, whereas the blank NPs without TPL loading did not induce the cell viability reduction (Fig. 3, Additional file 1: Fig. S6). In media at pH 7.4, TPL/NPs exhibited slightly higher inhibition of cellular proliferation than that with free TPL, and the IC_50_ values of TPL/NPs in both MDA-MB-231 and MCF-7 cells were close to that of free TPL. However, the TPL/NPs showed much higher cytotoxicity at all concentrations of TPL exposed to either of these culture media. Specifically, the average IC_50_ value of TPL/NPs in MCF-7 and MDA-MB-231 cells in a medium at pH 7.4 was 58.67 and 72.28 nM, respectively, whereas this was markedly reduced to 20.76 and 41.77 nM, respectively, in a medium at pH 7.4 with 10 mM GSH and was further reduced to 18.11 and 34.14 nM, respectively, in a medium at pH 5.8. The IC_50_ values of TPL/NPs under the medium of pH 5.8 + 10 mM GSH were the lowest among all the conditions tested and sharply decreased to 13.77 and 26.39 nM in MCF-7 and MDA-MB-231 cells, respectively (Fig. 3c). These results are consistent with the in vitro drug release profile, indicating that the pH-/redox-sensitive NPs facilitate drug release and efficiently kill tumor cells.

### TPL/NPs enhanced the cell apoptosis and cell cycle arrest

**Fig. 4:**
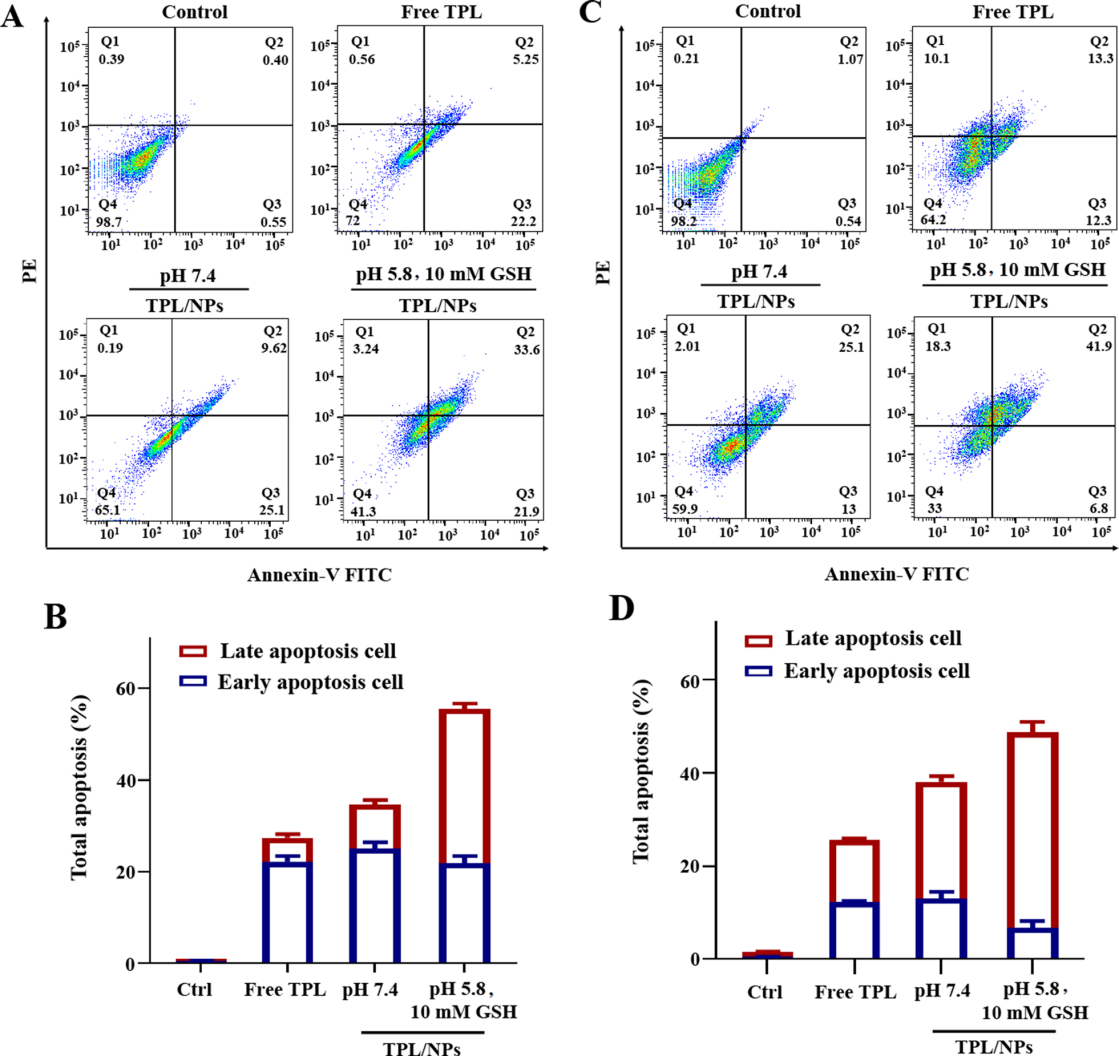
Representative images of Annexin V-FITC and PI staining of MDA-MB-231 (**a**) and MCF-7 (**c**) cells treated with TPL/NPs (20 nM) for 24 h. Quantification results of TPL/NPs after apoptosis induction in MDA-MB-231 (**b**) and MCF-7 (**d**) cells

Apoptosis is the main mechanism accounting for the anticancer activity of TPL [25]. Here, we further evaluated whether TPL/NPs would enhance apoptosis in both MDA-MB-231 and MCF-7 cells. To thoroughly mimic the acid and reductive tumor microenvironment, we induced apoptosis with TPL/NPs at pH 7.4 without GSH or at pH 5.8 with 10 mM GSH; apoptosis was analyzed via annexin V-fluorescein isothiocyanate (FITC)/propidium iodide (PI) staining. As Fig. 4 shows, with the equivalent TPL concentration of 20 nM in a 24 h incubation of MDA-MB-231 cells, TPL/NPs produced a higher rate of apoptosis than that of free TPL at each condition. Particularly, TPL/NPs in a culture medium at pH 5.8 with 10 mM GSH induced the highest rate of apoptosis compared with that in the other cultures. The rate of apoptosis induced by TPL/NPs at pH 5.8 with 10 mM GSH was 2- and 1.6-fold that induced by free TPL and TPL/NPs at pH 7.4, respectively. Moreover, the results in MCF-7 cells agreed with those in MDA-MB-231 cells.

**Fig. 5:**
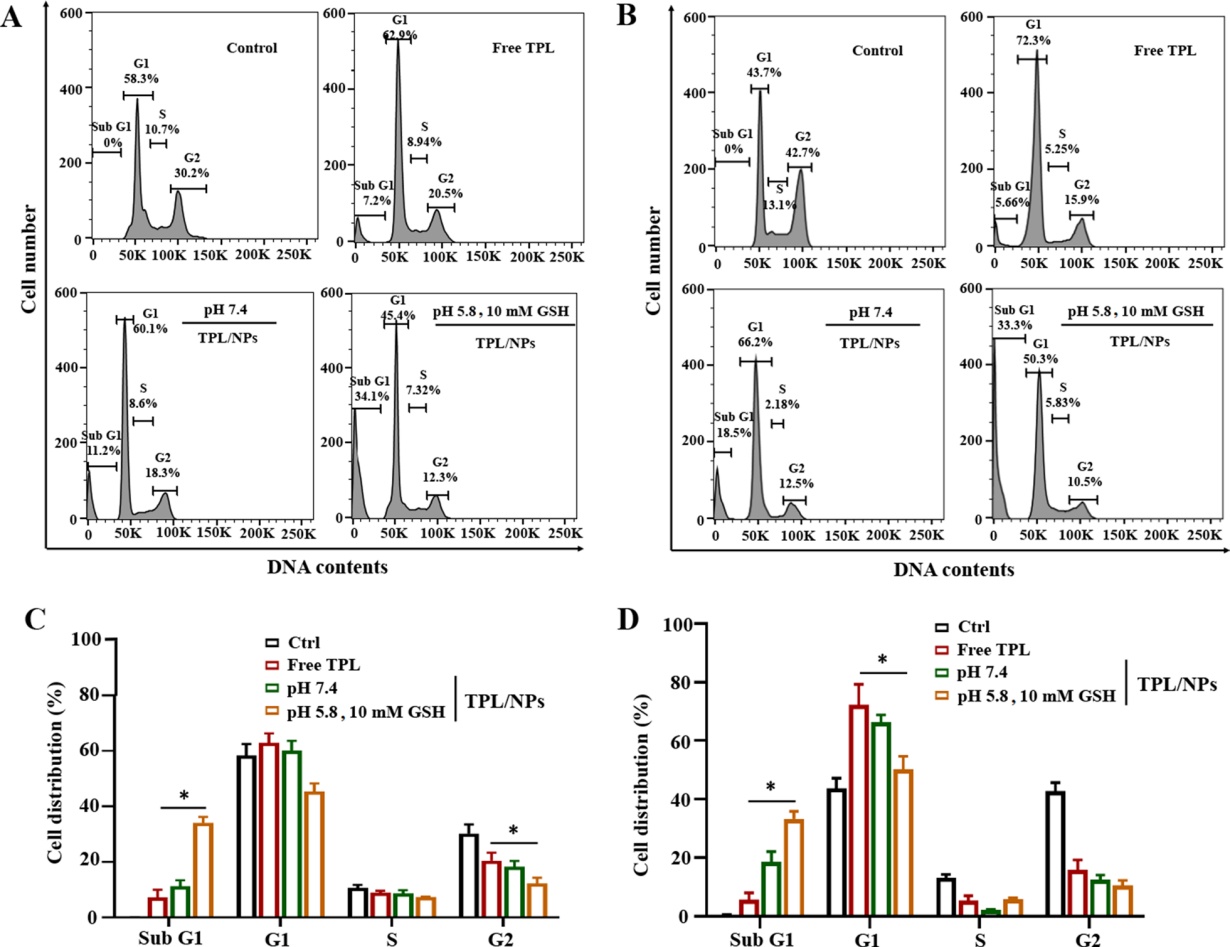
Representative cell cycle distribution histograms of MDA-MB-231 (**a**) and MCF-7 (**b**) cells treated with TPL/NPs for 24 h. Relative cell cycle distribution of Sub G1, G1, S, and G2 phases in MDA-MB-231 (**c**), and MCF-7 (**d**) cells

The cell cycle phase distribution was analyzed via flow cytometry (FCM) with PI staining, which measures the cellular DNA content. As Fig. 5 shows, after 24 h incubation of MDA-MB-231 cells with 10 nM TPL, a high proportion of cells were observed in the Sub G_0_/G_1_ phase, which indicated the occurrence of cell apoptosis. This was in accordance with a previous report concerning the TPL-mediated Sub G_1_ phase arrest [26]. Meanwhile, more MCF-7 and MDA-MB-231 cells were significantly arrested in the G_0_/G_1_ phase when treated with TPL/NPs compared with those treated with free TPL. Expectedly, the TPL/NPs in the medium at pH 5.8 + 10 mM GSH also induced a significantly higher G_0_/G_1_ population than that in the medium at pH 7.4.

In summary, TPL/NPs exhibited a stronger proapoptotic response and G_0_/G_1_ cell cycle arrest in both MDA-MB-231 and MCF-7 cells compared with those induced with free TPL. Given the stimuli-responsive drug release of TPL/NPs, both the cell apoptosis and cell cycle arrest of TPL/NPs exhibited significant enhancement in response to pH/redox culture medium, suggesting that TPL/NPs would have a beneficial anticancer effect in the tumor microenvironment.

### Cellular uptake study

**Fig. 6:**
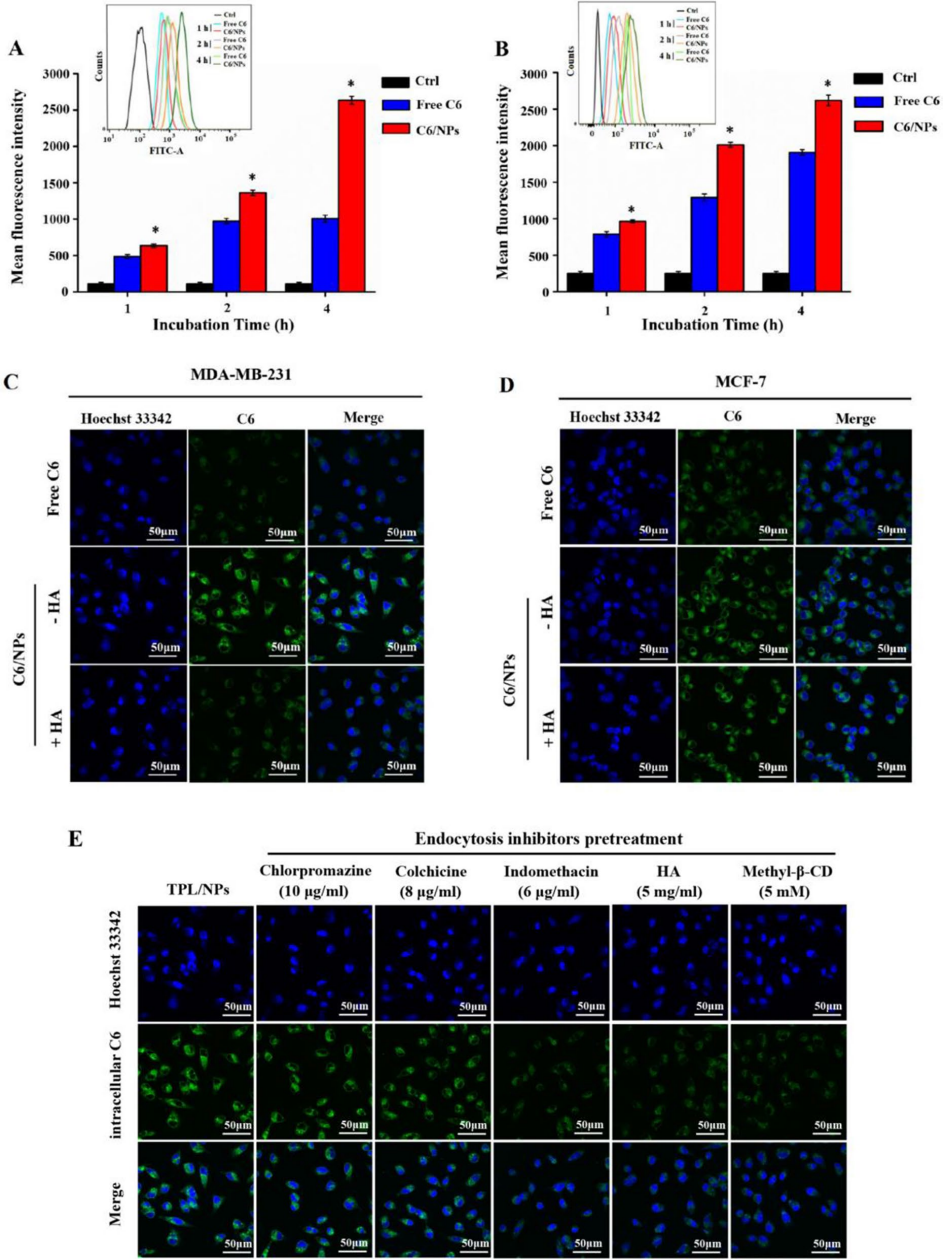
Intracellular fluorescence of MDA-MB-231 (**a**) and MCF-7 (**b**) cells determined by FCM following treatment with either free C6 or C6/NPs, respectively, for 1, 2, and 4 h. Representative cellular uptake images of MDA-MB-231 (**c**) and MCF-7 (**d**) cells after incubation with free C6 and C6/NPs for 4 h by CLSM observation. Images of cellular internalization of C6/NPs in MDA-MB-231 cells after pretreatment with endocytosis inhibitors (**e**). Note: *p < 0.05 C6/NPs versus free C6 group

To determine whether the TPL/NPs specifically enhanced cellular uptake of breast cancer cells, we used FCM to measure the uptake of Coumarin 6-loaded NPs (C6/NPs) in MDA-MB-231 and MCF-7 cells after treatment for 1, 2, and 4 h. After incubation with free C6 or C6/NPs, there was a time-dependent accumulation of free C6 and C6/NPs in both MDA-MB-231 and MCF-7 cells (Fig. 6a and b). Moreover, after co-incubation with cells, the cellular uptake of C6/NPs was significantly greater than that of free C6 at different time intervals, indicating enhanced cellular uptake of nanosized agents. The enhanced cellular uptake of C6/NPs was mediated by active transport in an energy-dependent manner (Additional file 1: Fig. S7a) rather than by the passive diffusion of free hydrophobic C6. Additionally, drug loading in NPs could avoid being easily pumped out of cells.

Furthermore, we used confocal laser scanning microscopy (CLSM) to observe the cellular uptake of C6/NPs both in MDA-MB-231 and MCF-7 cells (Fig. 6c and d). After treatment for 4 h, higher cellular internalization of C6 was found in cells treated with C6/NPs than that in cells treated with free C6. These data were consistent with FCM results, indicating that C6/NPs were effectively internalized by cells.

### Competitive uptake study

To investigate the CD44-targeted delivery of the TPL/NPs, the CD44-positve MDA-MB-231 breast cancer cell line and CD44-negative MCF-7 epithelial cell line were chosen (Additional file 1: Fig. S8) [27]. CLSM analysis (Fig. 6c and d) shows the intracellular uptake of C6/NPs in CD44-positive MDA-MB-231 cells and CD44-negative MCF-7 cells. The cell nuclei were stained with Hoechst 33,342, which exhibited strong blue fluorescence. Compared with that of the free C6 group, we observed a more intense fluorescence signal in MDA-MB-231 and MCF-7 cells when incubated with C6/NPs. However, when the C6/NPs group was pretreated with free HA for 1 h (C6/NPs + HA), the fluorescence expression was significantly decreased in MDA-MB-231 cells, which indicated that free HA blocked the CD44 receptor on the surface of MDA-MB-231 cells and inhibited internalization of C6/NPs. However, when the C6/NPs group was pretreated with 5 mg/mL of free HA for 1 h (C6/NPs + HA), the fluorescence expression was only partially reduced in MCF-7 cells. These results indicated that HA-modified NPs could specifically bind to the CD44 receptor on the surface of tumor cells and promote cellular uptake by active targeting.

Furthermore, we used FCM to assess the HA competition in CD44-positive MDA-MB-231 cells. The cells were pretreated with free HA followed by incubation with C6/NPs. This reduced the cellular accumulation of C6/NPs to 48.58% ± 3.7% (Additional file 1: Fig. S7b) and demonstrated that the cellular uptake of C6/NPs contributed to HA polymer-specific binding to CD44 receptors. The free HA competed with HA-conjugated NPs for CD44 receptors and inhibited cellular uptake of HA-conjugated NPs in cells.

### Endocytosis pathway

We utilized different endocytic inhibitors to identify the possible endocytotic pathways involved in the uptake of TPL/NPs, including chlorpromazine (clathrin-mediated), indomethacin (caveolae-mediated), methyl-β-cyclodextrin (cholesterol-dependent endocytosis), and colchicine (macropinocytosis inhibitor) [28]. As Fig. 6e shows, the internalization of C6/NPs was significantly decreased by pretreatment with indomethacin or methyl-β-cyclodextrin, indicating the process of internalization of C6/NPs was involved in caveolae-mediated and cholesterol-dependent endocytosis. By contrast, pretreatment with either chlorpromazine or colchicine produced a minimal effect on the cellular uptake of C6/NPs, which excluded the involvement of clathrin-mediated endocytosis and macropinocytosis.

### TPL/NPs inhibited MDA-MB-231 cell migration and invasion

**Fig. 7:**
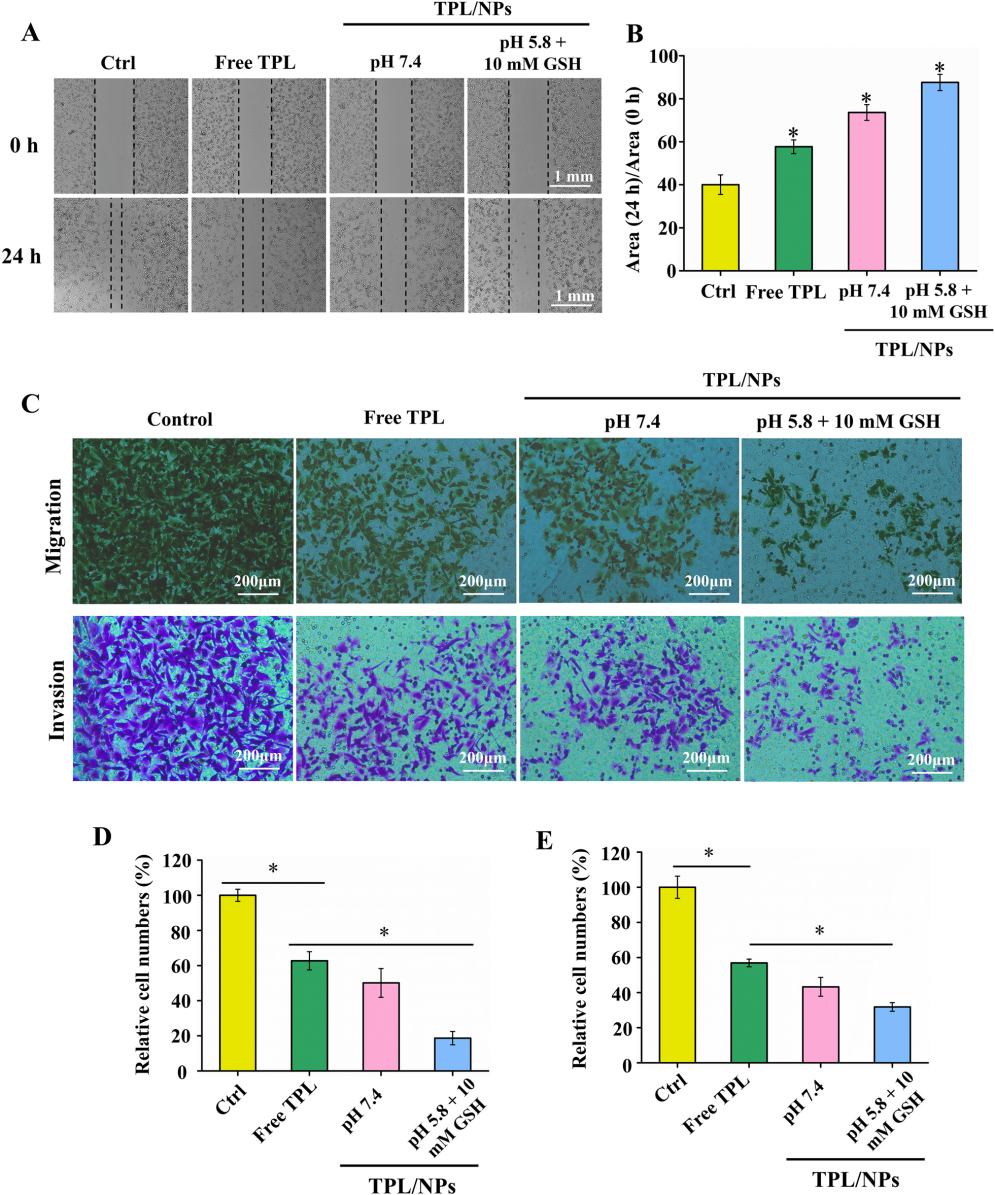
Effect of TPL/NPs on MDA-MB-231 cell migration and invasion. **a** Images of wound healing assays (magnification × 100). Cells seeded into 12-well cell culture plates and cultured to near confluency. The wounded monolayer was treated with PBS, free TPL, TPL/NPs in a medium at pH 7.4, and TPL/NPs in a medium at pH 5.8 with 10 mM GSH for 24 h. **b** Scratch area measured with ImageJ software. **c** Photographs of migration and invasion of MDA-MB-231 cells with different treatments. Percent of cell migration (**d**) and invasion (**e**)

The MDA-MB-231 cell line is a triple-negative breast cancer cell and possesses a high metastatic character [29]. We conducted a wound healing assay to evaluate whether TPL/NPs treatment could suppress the migration of MDA-MB-231 cells. The results showed that the wound gaps in the TPL/NPs-treated groups were significantly wider than either those in the untreated groups or those in the free TPL treatment group, indicating that TPL/NPs could more effectively inhibit the migration of MDA-MB-231 cells compared with free TPL (Fig. 7a and b). Particularly, the inhibition of migration after treatment with TPL/NPs in a medium at pH 5.8 with 10 mM GSH was remarkably higher than that in the other cultures.

The antimetastatic effect of TPL/NPs was also evaluated by Transwell experiments. The results were consistent with those of the wound healing assay and showed that TPL/NPs effectively suppressed cell migration. Only a few MDA-MB-231 cells migrated from the upper chamber to the lower chamber of the Transwell following treatment with TPL/NPs in a medium at pH 5.8 + 10 mM GSH, indicated that TPL/NPs significantly inhibited the longitudinal motility of MDA-MB-231 cells (Fig. 7c and d).

To study the effect of TPL/NPs on cell invasion, MDA-MB-231 cells were treated with either free TPL or TPL/NPs and then allowed to invade Matrigel-coated Transwells for 24 h. The number of invasive cells was decreased by various TPL formulas (Fig. 7c and e). Compared with results in the untreated group, free TPL, TPL/NPs at pH 7.4, and TPL/NPs at pH 5.8 + 10 mM GSH inhibited invasion by approximately 45%, 52%, and 67%, respectively. These results clearly demonstrated that TPL/NPs could strongly suppress MDA-MB-231 cell migration and invasion and that this effect was sensitive to the pH/redox conditions. The above results implied that TPL/NPs had antimetastatic potential.

### In vivo biodistribution of HA-VE/PBAEss NPs

**Fig. 8:**
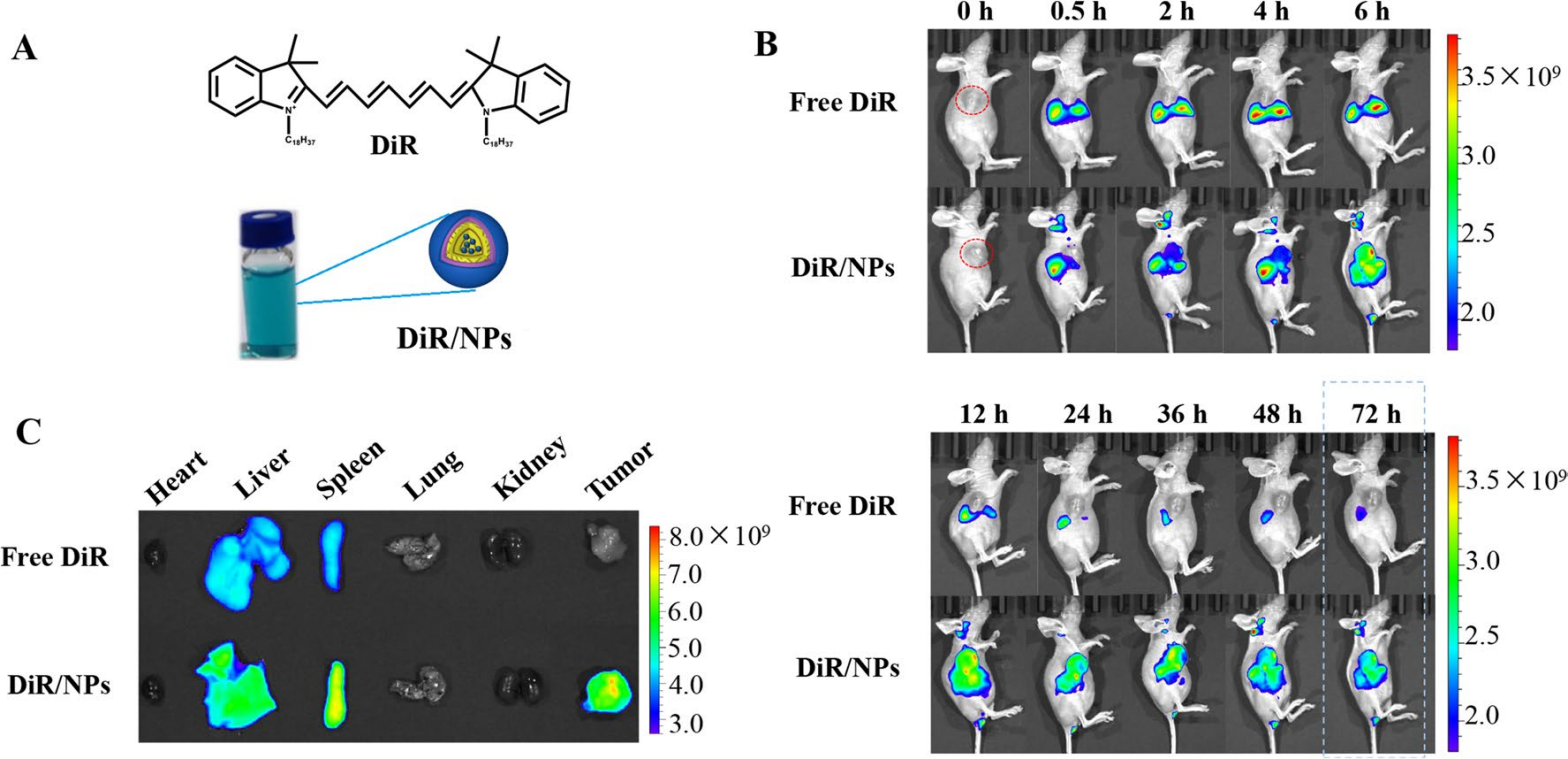
Tumor-targeting profiles of HA-VE/PBAEss NPs in vivo by IVIS spectrum. **a** Schematic diagram showing the preparation of the fluorescent NPs using DiR dye loaded in HA-VE/PBAEss NPs using a similar approach as that used with TPL/NPs. **b** Fluorescent distribution of DiR/NPs in vivo after vein injection at predetermined time intervals, i.e., 0, 0.5, 2, 4, 6, 12, 24, 36, 48, and 72 h after injection. **c** Ex vivo fluorescence images of dissected organs of the mice 72 h postinjection

The tumor-targeting capacity of HA-VE/PBAEss NPs was further investigated in 4T1 tumor-bearing nude mice. To label the HA-VE/PBAEss NPs with fluorescence, 1,10-dioctadecyltetramethyl indotricarbocyanine iodide (DiR) was first encapsulated into NPs using a similar approach as TPL, as shown in Fig. 8a. When the tumor volume reached approximately 1000 mm^3^, the mice were intravenously administered with free DiR and DiR/NPs. The mice were imaged using the IVIS® Spectrum scanner during 72 h postinjection period (Fig. 8b). At 72 h, the mice were euthanized, and the main organs as well as the tumors were harvested and visualized ex vivo (Fig. 8c). After administration of free DiR, the near-infrared (NIR) fluorescence in vivo was sharply attenuated, and limited fluorescence could be found in tumor tissue, indicating rapid body clearance and poor tumor accumulation capacity. However, much more fluorescence over a prolonged period can be found in mice treated with DiR/NPs. Particularly, significant tumor accumulation of DiR/NPs could be observed. Even after 72 h postinjection, strong fluorescence remained in the tumor tissue compared with the inconspicuous fluorescence in the tumor in the free DiR group. This indicated that vesicles composed of HA-VE/PBAEss copolymers had accumulated in the tumor, which would contribute to the prolonged circulation, EPR effect, and tumor-targeting mediated by HA and CD44 interaction. Taken together, these results indicated that the in vivo extended circulation and tumor accumulation of TPL can be efficiently enhanced by encapsulation within HA-VE/PBAEss NPs.

### Inhibition of primary tumor growth and metastasis in vivo

**Fig. 9:**
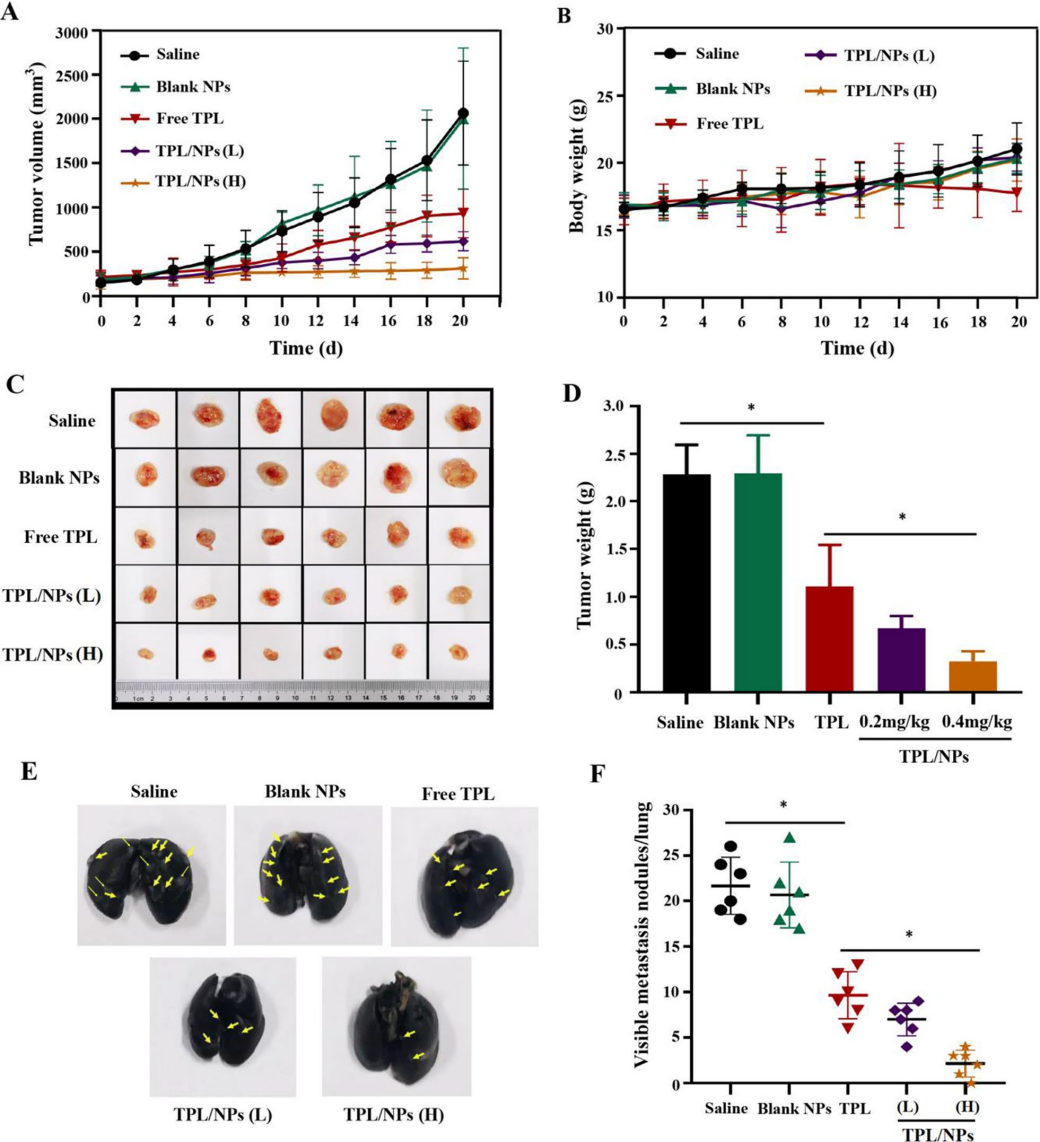
In vivo suppression activities of TPL/NPs on primary tumors and lung metastasis in the 4T1 xenograft model. **a** Growth curve of the 4T1 xenografts in BALB/c mice treated with saline, blank NPs, free TPL, TPL/NPs (L), and TPL/NPs (H) for 20 days. Data are presented as the mean ± standard error (SD, n = 6 mice). **b** Body weight as a function of time. **c** Picture of excised tumors at the end of the experiment. **d** Tumor weight at the end of the experiment. **e** Representative photograph of the 4T1 mouse lungs with the India ink-stained whole lungs at the end of the experiment. Representative tumors are marked by arrows. **f** Semiquantitative analysis of metastatic nodules in lungs at the end of the experiment when treated with saline, blank NPs, free TPL, TPL/NPs (L), and TPL/NPs (H) for 20 days. Note: *p < 0.05

The combination of tumor-homing properties and dual-stimuli-triggered tumor release profiles of TPL/NPs indicated that these would work efficiently in chemotherapy. Therefore, we established a 4T1 subcutaneous tumor-bearing mouse model to further evaluate the in vivo efficacy in suppressing primary tumor growth and distant metastasis. After treatment with different administrations for 20 days, the tumor growth curves (Fig. 9a) and body weight (Fig. 9b) were measured every 2 days. As shown, the blank HA-VE/PBAEss NPs had a similar effect as the saline control group and did not affect tumor growth or body weight. However, mice treated with TPL formulations exhibited greater antitumor activity compared with that of the saline group. The tumor volume inhibition rates for TPL, TPL/NPs (0.2 mg/kg, L), and TPL/NPs (0.4 mg/kg, H) were 54.86%, 70.10%, and 84.76%, respectively. Particularly, the TPL/NPs at either 0.2 or 0.4 mg/kg induced higher tumor growth inhibition efficacy than that of free TPL at 0.4 mg/kg. The TPL/NPs (H) nearly completely inhibited the growth of the tumor-bearing tissue. Meanwhile, after a successive administration of free TPL for 14 days, mice exhibited a decline in body weight, indicating the potential side effects. Nevertheless, after loading in NPs, TPL exhibited a higher biosafety profile than that of free TPL. The differences among groups on inhibition of growth of the primary tumor are shown in Fig. 9c. At the experimental end point, the tumor weight in each group had the same trend as the result of tumor volume (Fig. 9d). These results demonstrated that TPL/NPs (H) with 0.4 mg/kg TPL possessed induced the best curative effect on primary tumor and with no side effects.

4T1 subcutaneous tumors have been demonstrated to be prone to inducing pulmonary metastasis. In view of the cell migration and invasion inhibition effects in vitro, the antimetastatic ability of TPL/NPs in vivo was also evaluated. After perfusing the lungs with ink gelatin solution, the pulmonary metastatic nodules were visible. A large number of metastatic nodules were observed in the lungs of the saline and blank NPs groups (Fig. 9e). However, compared with the saline group, the number of pulmonary metastatic nodules in mice treated with TPL, TPL/NPs (L), and TPL/NPs (H) decreased by 55.38%, 67.70%, and 89.99%, respectively (Fig. 9f). The number of pulmonary metastatic nodules in the TPL/NPs (H) group was markedly reduced compared with that in other conditions. These results corroborated that TPL/NPs could efficiently suppress primary tumor growth and pulmonary metastasis.

### Potential antitumor and antimetastatic mechanisms

**Fig. 10:**
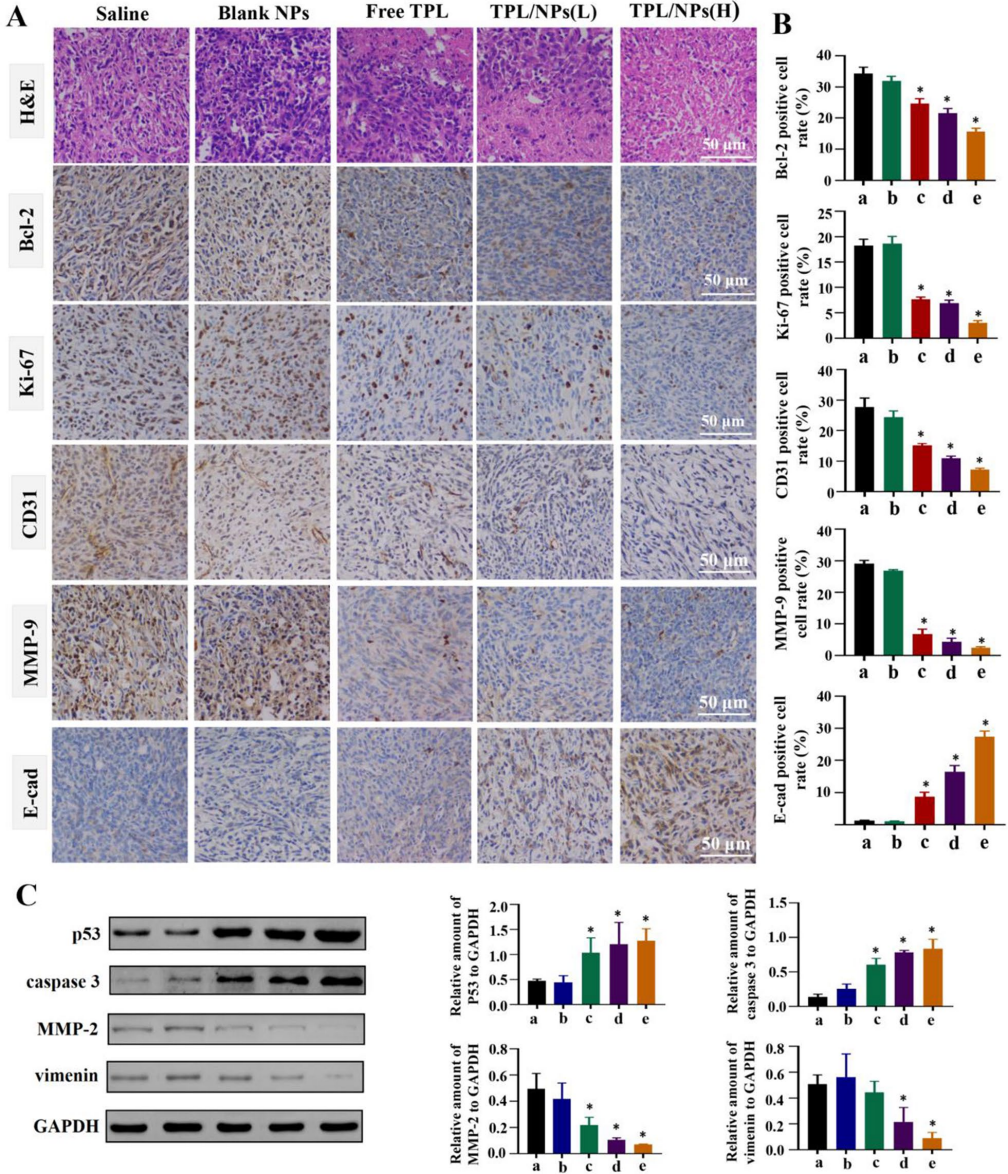
Histological staining and western blot analysis of representative proteins in tumor sections derived from mice treated with saline, free TPL, blank NPs, TPL/NPs (L), and TPL/NPs (H). **a** H&E staining and immunohistochemistry staining of Bcl-2, Ki-67, CD31, MMP-9, and E-cad; **b** Statistical analysis for the percentage of Bcl-2, Ki-67, CD31, MMP-9, and E-cad expression in the tumor sections by immunohistochemistry staining. The expression of EMT-relative proteins (MMP-2 and vimentin) and mitochondrial apoptosis pathway-relative proteins (p53 and caspase 3) evaluated by western blotting (**c**). Semiquantification of the expression level of these proteins (**d**). Note: *p < 0.05

To further investigate the mechanisms of proliferation and metastasis inhibition of TPL/NPs in vivo, the tumor tissues were assessed by hematoxylin and eosin (H&E) and immunohistochemical staining (Fig. 10a). The H&E images indicated that the TPL/NPs induced limited cell proliferation but caused severe apoptotic damage, even necrocytosis, in the tumor compared with effects in cells treated with either free TPL or blank NPs. This result was also confirmed by the immunohistochemical staining using Bcl-2 and Ki-67 antibodies. In the TPL/NPs (H) group, the number of brownish-yellow points representing positive cells was far fewer than that in other test conditions. In our previous study, we demonstrated that TPL could significantly suppress angiogenesis, which helped to block tumor growth. Here, the expression of CD31 was significantly downregulated in TPL/NPs (H) group compared with that in other groups, indicating the higher antiangiogenesis activity of TPL/NPs. Moreover, the expression of matrix metalloproteinase-9 (MMP-9) in the tumor tissue has a close relationship with tumor invasion and metastasis. As shown, the expression of MMP-9 was uniform over the whole slice of the saline group, whereas the lowest expression of MMP-9 was observed with treatment of TPL/NPs (H). E-cadherin (E-cad) is an essential epithelial-to-mesenchymal transition (EMT)-relative protein that plays a pivotal role in the invasion and metastasis of several epithelial malignant tumors. Invasion of surrounding tissues and metastasis have been proposed to be initiated following the loss of E-cad [30]. Expectedly, low expression of E-cad could be found in the saline group, whereas the E-cad expression was markedly increased in the TPL/NPs (H) group. The downregulated MMP-9 and upregulated E-cad expression indicated that the metastasis process was significantly restrained following treatment with TPL/NPs (Fig. 10b). Similar results were also observed in histological staining images of lung tissues (Additional file 1: Fig. S9). The generation of pulmonary metastatic nodules indicated that cell proliferation and blood vessel and cell migration in the lung tissues of the saline group was extremely active. However, treatment with TPL/NPs could effectively reduce the abnormal upregulation of the expression of representative proteins, including Bcl-2, Ki-67, CD31, MMP-9, and E-cad, in the lung tissue. These results were consistent with the conclusion obtained from the tumor tissue of immunohistochemical analysis.

Furthermore, to validate the regulation effect on tumor growth and metastasis of TPL/NPs, western blotting was also performed to assess the expression of signaling proteins involved in apoptosis and metastasis, including p53, caspase 3, MMP-2, and vimentin. As shown in Fig. 10c and d, treatment with TPL/NPs significantly upregulated the protein levels of p53 and caspase 3 compared with those in either the control or free TPL groups. Thus, combined with the regulation of Bcl-2, caspase 3, and p53, the data demonstrated that TPL/NPs significantly induced tumor apoptosis mediated by the mitochondrial apoptotic pathway. Additionally, the representative proteins, in the tumor EMT process including MMP-2 and vimentin, were evaluated via western blotting. Expectedly, the levels of MMP-2 and vimentin were markedly decreased in the TPL/NPs groups (Fig. 10d). In view of the regulation of E-cad, MMP-9, MMP-2, and vimentin, the results showed that TPL/NPs effectively suppressed tumor metastatic effects in vivo.

**Fig. 11:**
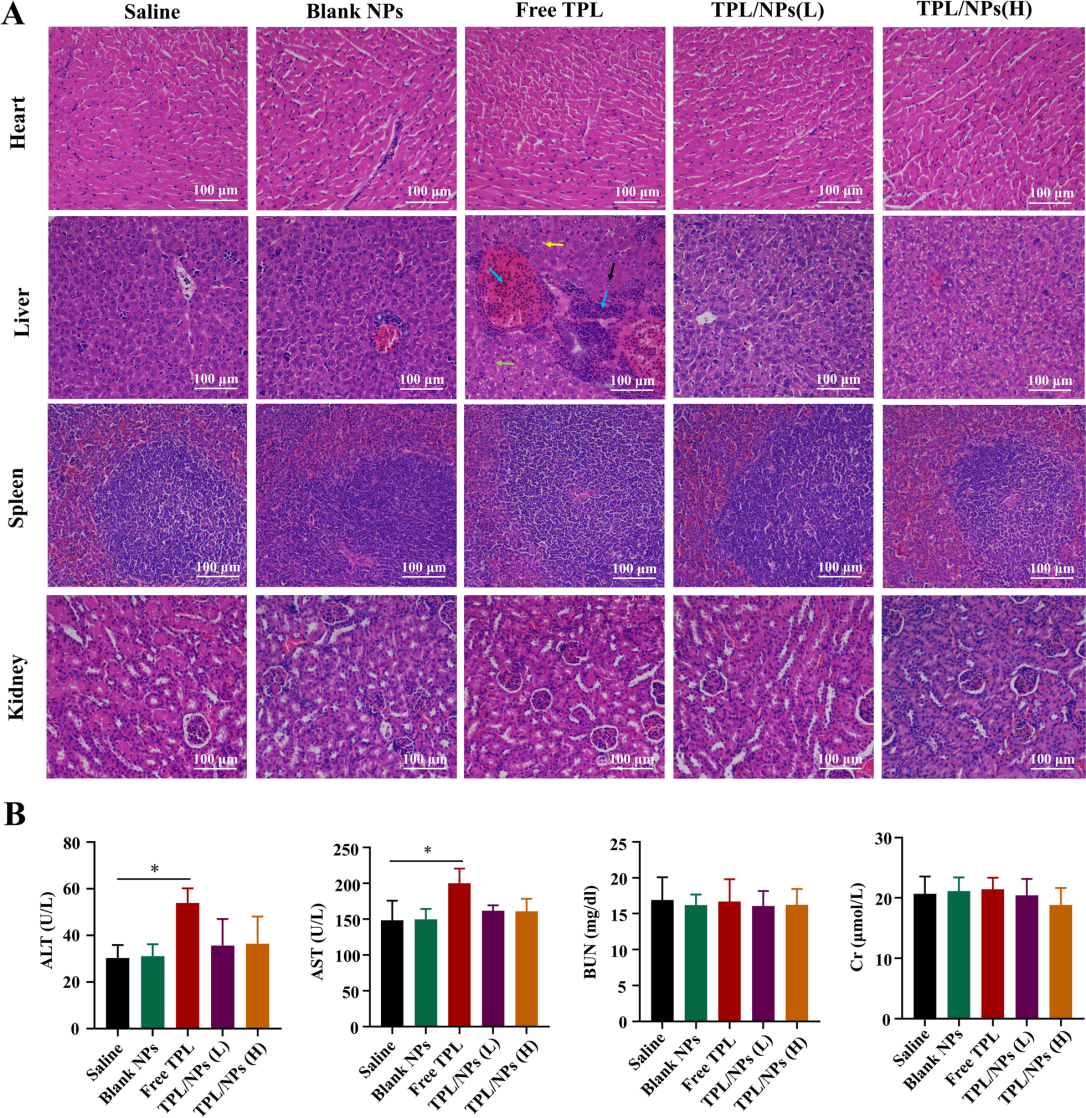
Systemic toxicity in tumor-bearing mice treated with saline, free TPL, blank NPs, TPL/NPs (L), and TPL/NPs (H). **a** Histopathological changes in heart, liver, spleen, and kidney tissues. Note: poor cytoplasm and light staining (black arrow), nucleus pyknosis and deep staining (green arrow), fat vacuoles (yellow arrow), inflammatory cell infiltration (blue arrow). **b** Levels of ALT, AST, BUN, and Cr in mouse serum at the therapeutic end point of different treatments. Note: *p < 0.05

Although treatment with TPL/NPs produced outstanding antitumor growth and antimetastatic effects, whether this would result in side effects remains unclear, in view of the acknowledged severe side effects of TPL. Thus, we evaluated the histological images of the main organs using H&E staining and several blood biochemical indexes. Compared with those in the saline group, free TPL-treated mice showed histopathological changes in liver tissue, characterized by fat vacuoles, inflammatory cell infiltration, poor cytoplasm and light staining, nucleus pyknosis and deep staining (Fig. 11a). This indicated that liver injury had been caused by free TPL administration. However, the severity of histopathological lesions in TPL/NPs groups was less than that in the free TPL group. Furthermore, significant increases in alanine aminotransferase (ALT) and aspirate aminotransferase (AST) levels in blood were observed in mice serum treated with 0.4 mg/kg of the free TPL compared with those in the saline group (Fig. 11b). By contrast, the administration of TPL/NPs with each dosage did not lead to significant changes in the levels of these indexes. There were no significant differences in the levels of urea nitrogen (BUN) or creatinine (Cr) between the groups.

## Discussion

Presently, surgery and chemotherapy are the main treatment methods for breast cancer. However, because of the nonspecificity of tumors and drug-related toxicity to nontargeted tissues and organs, the clinical efficacy of systemic therapy is usually affected. Additionally, breast cancer metastasis is one of the main causes of death due to cancer in female patients. Therefore, methods to improve the efficacy of chemotherapeutic drugs and overcome tumor metastasis could significantly improve the prognosis of patients with breast cancer.

TPL has strong pharmacological effects, but the toxic effects on organs including the liver, kidney, skin, gastrointestinal tract, heart, and reproductive system limit the therapeutic window [31]. In this study, we attempted to reduce the toxicity of TPL and enhance the antitumor inhibition and metastatic activity. The TPL/NPs is a highly effective and low toxic tumor therapy that could solve the systemic toxicity of TPL. This may be attributed to the enhanced accumulation of NPs at the tumor and redox- and pH-responsive targeting within tumor cells via HA and CD44.

Furthermore, our work demonstrated that the administration of both the free TPL and TPL/NPs efficiently reduced the expression levels of Bcl-2 and increased the expression levels of p53 and caspase 3, which are crucial proteins in the apoptosis signaling pathway. More interestingly, our data further demonstrated a stronger regulation of targeting efficacy of the TPL/NPs compared with that of the free drug. As the most essential marker for EMT, E-cad has been reported to be responsible for intercellular connections and polarity [32]. Expectedly, the expression of E-cad was significantly upregulated in the free TPL- and TPL/NPs-treated groups. However, TPL/NPs directly regulated EMT progression by decreasing vimentin expression. MMPs are a family of zinc-dependent endopeptidases and are the main proteases that invade and degrade basement membranes and the extracellular matrix. As a previous study reported, inhibiting MMPs could significantly reduce invasiveness [33]. MMP-2 and MMP-9 are the key members of the MMP family and can cleave gelatin, collagens, elastin, and vitronectin and are necessary for cell invasion and migration [34]. Our results show that TPL/NPs inhibit the expression of MMP-2 and MMP-9. In summary, our results indicate that TPL/NPs can significantly inhibit cell apoptosis, proliferation, and invasion and thereby exhibit an excellent antimetastatic effect in vivo.

## Conclusion

In this study, we developed a multifunctional nanocarrier via self-assembly of HA and VE together with PBAEss polymers to enhance the TPL-based inhibition of tumor growth and lung metastasis. This nanosystem possesses integrated advantages including tumor-targeting mediated via the CD44 receptor, proton sponge effect in response to acidic endosome pH, and GSH-responsive drug burst release as well as the combined anticancer and antimetastasis effect of TPL. In vitro cellular experiments confirmed that the enhanced cellular proliferation inhibition of TPL/NPs against both MCF-7 and MDA-MB-231 cells was related to the upregulation of cell apoptosis and cell cycle arrest. Additionally, TPL/NPs exhibited prominent antimigration and antiinvasion activities in MDA-MB-231 cells. Furthermore, because of the superior tumor accumulation capacity, TPL/NPs efficiently inhibited the growth and lung metastasis of breast tumor cells in the 4T1 carcinoma xenograft mouse model and exhibited lower systemic toxicities than free TPL. This work revealed that TPL/NPs are promising candidates in halting breast cancer progression and metastasis while minimizing systemic toxicity.

## Methods

### Chemicals and reagents

TPL was obtained from Chengdu DeSiTe Biological Technology Co., Ltd (Chengdu, China). HA (molecular weight 8 kDa) was acquired from Freda Biochem Co., Ltd. (Shandong, China). DiR was obtained from Mellon, Biological Technology Co., Ltd. (Dalian, China). Hoechst 33,342 was provided by Suzhou Yuheng Biotechnology Co., Ltd. (Suzhou, China). HDD and BACy were purchased from Macklin Reagent Co., Ltd. (Shanghai, China). VE (purity ≥ 98%), EDC, DMAP, dimethyl sulfoxide (DMSO), and MTT were obtained from Macklin Reagent Co. Ltd. (Shanghai, China). DMEM, fetal bovine serum (FBS), horse serum (HS), PBS, penicillin–streptomycin, and 0.25% (w/v) trypsin containing 1 mM EDTA were purchased from Invitrogen (Carlsbad, CA, USA). FITC antihuman CD44 was purchased from 4A Biotech Co., Ltd. BCA protein quantitative detection kit was supplied by MultiScience (Lianke) Biotech Co., Ltd. (Hangzhou, China). Other organic solvents or reagents were of analytic grade and used as received.

### Cell culture

MDA-MB-231, MCF-7, and 4T1 cells were purchased from American Type Culture Collection (Manassas, USA). PC-12 cells were obtained from the Kunming Institute of Zoology (Kunming, China). DMEM containing 10% FBS and 1% penicillin–streptomycin was used to culture MDA-MB-231, MCF-7, and 4T1 breast cancer cells. DMEM supplemented with 10% HS, 5% FBS, and 1% penicillin–streptomycin was used to culture PC-12 cells. All cells were incubated at 37 °C in a humidified atmosphere with 5% CO_2_.

### Animal feeding

Female BALB/c nude mice (4 weeks old; 14–16 g) were obtained from SPF Biotechnology Co., Ltd. (Beijing, China) and kept in an animal care facility for a 12 h light/dark cycle. All animals were acclimated for at least 7 days before the experiments began and were given a fresh diet and free drinking water. The animal experiment ethics certification was obtained from Chengdu University of Traditional Chinese Medicine (permit CDU2019S121). All animal experiments were conducted according to university guidelines. Animal welfare was guaranteed in all animal experiments.

### Synthesis of HA-VE and PBAEss copolymers

HA-VE conjugates were synthesized by an esterification reaction of HA with VE [35]. Briefly, VE (2 mmol) and EDC (3 mmol) were dissolved in 20 mL of anhydrous DMSO and stirred for 2 h to activate the carboxyl group. Next, HA (2 mmol) and DMAP (2 mmol) were added into the mixture, and the reaction continued at 30 °C for 48 h with stirring. Then, the HA-VE mixture was successively dialyzed against deionized water using a dialysis tube (molecular weight cut-off [MWCO] 1000 kDa) for 2 days to remove unreacted material. The HA-VE was then lyophilized and stored at − 20 °C. The structure of HA-VE was characterized using ^1^H NMR [36].

We used a Michael-type step polymerization to synthesize the PBAEss copolymer [36]. Briefly, 4-amino-1-butanol (5 mmol), HDD (2.5 mmol), and BACy (2.5 mmol) were dissolved using 10 mL DMSO with continuous stirring for 24 h at 90 °C. This material was then dialyzed (MWCO 5000 kDa) first in DMSO and subsequently in PBS (pH 7.4) to remove unreacted materials. Finally, PBAEss was obtained after freeze drying and stored at − 20 °C. ^1^H NMR was used to characterize the structure of PBAEss, and GPC was used to measure the molecular weights of the synthesized PBAEss copolymers [37].

### Preparation and characterization of TPL/NPs

TPL encapsulated in TPL/NPs composed by HA-VE and PBAEss copolymers (TPL/NPs) was prepared by a nanoprecipitation method. In short, PBAEss and TPL were dissolved in 2 mL methanol and dispersed via sonication at 90 W for 60 s. Then, HA-VE was dissolved in 10 mL PBS (pH 7.4) with mild stirring for 5 min. The mixture of organic solvent (containing TPL and PBAEss) was then dropped in an aqueous phase (PBS, pH 7.4, containing HA-VE) with stirring for 4 h at 30 °C to evaporate the residual methanol. Finally, we used a 0.45 μm microporous membrane to remove free TPL drugs. The obtained TPL/NPs sample was stored at 4 °C.

The characterization of TPL/NPs including size distribution, surface charge, morphology, drug loading capacity, drug release, and hemolytic and serum stability were implemented on the basis of the previous studies and described in the Supplementary Material.

### Redox- and pH-responsive capability of TPL/NPs

The TPL/NPs were dispersed in PBS (pH 7.4) with 10 mM of GSH over 24 h or within acidic environments at pH 5.8 over 24 h at a final diluted material concentration of 1 mg/mL with stirring at 100 rpm. After designated time intervals (0, 2, 4, 6, and 24 h), the mean particle sizes of the TPL/NPs were measured by a Zetasizer Nano ZS 90. Meanwhile, any morphological changes of TPL/NPs in response to either acid buffer (pH 5.8) or reductive buffer (10 mM GSH) were assessed via TEM observation.

### Cytotoxicity of TPL/NPs against breast cancer cells

MDA-MB-231 and MCF-7 human breast cells were seeded into 96-well plates at a density of 3 × 10^3^ cells/well for 24 h [38, 39]. MDA-MB-231 and MCF-7 cells were then given free TPL, blank NPs and TPL/NPs at various concentrations (0–160 nmol/L) in different culture media (pH 7.4; pH 7.4 + 10 mM GSH; pH 5.8; and pH 5.8 + 10 mM GSH) for 48 h. Cells treated with 0.1% DMSO served as the negative control. The MTT assay was used to measure cell viability, according to the manufacturer’s protocol.

### Apoptosis analysis via FCM

Cell apoptosis induced by various TPL formulas was detected by an annexin V-FITC/PI detection kit (Biovision, USA). MDA-MB-231 and MCF-7 cells (6 × 10^4^ cells/well) were seeded in six-well plates and then treated with free TPL and TPL/NPs at equivalent concentrations of 20 nM TPL in different media (pH 7.4 and pH 5.8 + 10 mM GSH, respectively) for 24 h. Cells were harvested with EDTA-free trypsin and stained with Annexin V-FITC and PI according to the manufacturer’s instructions. Finally, each sample was analyzed by using FCM (BD Biosciences, California, USA).

### Cell cycle analysis

Seeded MDA-MB-231 and MCF-7 cells (5 × 10^4^ cells/well) were cultured in an FBS-free medium for 24 h for cell cycle synchronization. Cells were then treated with free TPL and TPL/NPs with the equivalent concentrations of TPL at 10 nM in different media (pH 7.4 and pH 5.8 + 10 mM GSH, respectively) for 24 h. The cells were trypsinized, washed with PBS, and then fixed in cold 70% ethanol at 4 °C for 24 h. Cells were collected by centrifugation and stained with 5 μL PI (Life Technologies, USA) for 10 min. Cell samples were assessed using FCM, and the cell distribution of single-cell suspension was analyzed using FlowJo V10 software (version 3.0, USA).

### Cellular uptake study

To take advantage of the HA-CD44 interaction-mediated intracellular uptake, the expression level of CD44 receptors in MDA-MB-231, MCF-7, and P12 cells, which represented the high, low, and scarce expression of CD44 receptors, was detected via the immunofluorescence method, as detailed in the Supplementary Material.

Because of the positive expression of the CD44 receptor in MDA-MB-231 and MCF-7 cells, the cellular uptake of HA-VE/PBAEss NPs in cells was determined quantitatively and qualitatively. C6 was primarily loaded in HA-VE/PBAEss NPs based on a similar preparation approach as a fluorescent label. C6/NPs were then co-cultured with MDA-MB-231 and MCF-7 cells to investigate the cellular uptake behavior of NPs. Briefly, MDA-MB-231 and MCF-7 cells were respectively seeded into the six-well plate at a density of 2 × 10^5^ cells/well to adhere for 24 h. Then, the culture medium was replaced with a serum-free fresh medium containing free C6 or C6/NPs at an equivalent C6 concentration of 100 ng/mL. After incubation for 1, 2, and 4 h, the medium was removed and the cells were washed with cold PBS. Cells were collected by trypsinization and resuspended in PBS for FCM. The data presented were based on the mean fluorescence signal for 10,000 cells collected.

Additionally, CLSM was used to present the cellular uptake of NPs. MDA-MB-231 and MCF-7 cells were first seeded into 35 mm glass-bottom dishes for 24 h. Cells were then incubated with free C6 or C6/NPs at the equivalent C6 concentration of 100 ng/mL for 4 h. Finally, cells were washed with PBS and fixed in cold 4% paraformaldehyde for 10 min. Cell nuclei were stained with Hoechst 33,342 for 10 min and then observed by CLSM.

Because of the high expression of the CD44 receptor in MDA-MB-231 cells, the intracellular mechanisms of HA-VE/PBAEss NPs in MDA-MB-231 cells were evaluated using several endocytosis inhibitors. MDA-MB-231 cells were cultured in six-well plates at a density of 2 × 10^6^ cells/well. After overnight incubation, 1 mL medium containing several inhibitor agents, including 10 μg/mL chlorpromazine, 6 μg/mL indomethacin, 8 μg/mL colchicine, 5 mM methyl-β-cyclodextrin, and 5 mg/mL HA, was used to replace the original medium for 1 h. C6/NPs at a C6 concentration of 100 ng/mL then were co-cultured with cells for another 4 h. The intracellular fluorescence profiles were determined via CLSM and FCM as described above.

### Wound healing assay

MDA-MB-231 cells were seeded into a six-well plate and cultured to 80%–100% confluence. The cell monolayers were carefully damaged with a sterile toothpick and washed with PBS three times. The wounded cell monolayers were then treated with free TPL or TPL/NPs (5 nM) for 24 h, whereas the control group received the medium solution for the period. The cell distribution at the scratch zone was observed using a microscope. The scratch area was obtained by ImageJ software, and the percentage of wound closure was evaluated as the parameter of wound closure degree.

### Transwell migration and invasion assay

Cell migration and invasion assays in MDA-MB-231 cells were performed using a 24-well Transwell chamber as described previously. The upper and lower sides of the Transwell membrane (8 µm pores) were precoated with 0.1% collagen in the cell migration assay. In the cell invasion assay, the upper and lower sides of the membrane were precoated with 100 µL Matrigel (20% in blank medium). Briefly, MDA-MB-231 cells were seeded into the Transwell chambers at a density of 5 × 10^4^ cells/well with a low-serum medium. Cells were treated with free TPL or TPL/NPs for 24 h at 37 ℃, and the cells on the upper surface of the Transwell membrane were then removed using cotton swabs. Next, the Transwell membranes were fixed with 4% paraformaldehyde for 15 min and stained with Hoechst 33,342 (10 µg/mL) for 15 min. Finally, the membranes were mounted on microscope slides and images were captured using a fluorescence inverted microscope and a charge-coupled device camera (AxioCam HRC, Carl Zeiss, Oberkochen, Germany). Cell migration and invasion were quantified by counting the number of cells per insert using ImageJ software (National Institutes of Health, Bethesda, USA).

### Tumor accumulation imaging in vivo and ex vivo

The tumor accumulation profile of NPs was related to the tumor suppression effects and toxicity on normal organs. To track the biodistribution of NPs in vivo, DiR, a near-infrared fluorescence dye, was applied to replace TPL and loaded in NPs. Tumor-bearing mice were established by subcutaneous injection of 1 × 10^6^ 4T1 cells in 100 μL PBS containing 50% Matrigel on the right axilla of nude mice. When the tumor volume reached approximately 1000 mm^3^, the tumor-bearing mice were randomly divided into two groups; free DiR and DiR/NPs (with the equivalent DiR amount of 0.05 mg/kg) were administrated via tail vein injection. Images were obtained using Caliper LifeSciences LIVIS® Lumina Series (PerkinElmer, MA, USA) at an excitation wavelength of 745 nm and an emission wavelength of 800 nm. Fluorescence images were collected at 0, 0.5, 2, 4, 6, 12, 24, 36, 48, and 72 h postinjection. At 72 h postinjection, the mice were euthanized to obtain the tumor and heart, liver, spleen, lung, and kidney tissues. The data were analyzed using Living Image Version 4.5 software (PerkinElmer, MA, USA).

### In vivo antitumor and antimetastatic effect

The breast cancer model was established through subcutaneous flank inoculation of 1 × 10^6^ 4T1 cells in 100 μL PBS containing 50% Matrigel on BALB/c female mice. When the tumor volume reached 100–200 mm^3^, the mice were treated with saline (control), blank NPs, free TPL (0.4 mg/kg), TPL/NPs (0.2 mg/kg, L), and TPL/NPs (0.4 mg/kg, H) every other day via tail vein injection (n = 6). The tumor size was measured with calipers every other day, and tumor volumes were calculated according to the formula V = length × width^2^/2. Mouse body weights were monitored during the experimental period. After day 21, the mice were euthanized through an overdose of anesthetic (pelltobarbitalum natricum), and the tumors and the main organs (including the heart, liver, spleen, lung, and kidney) of mice were obtained and photographed. The tumors and the main organs were weighed and fixed with 4% (v/v) formalin neutral buffer solution and sectioned into 6 µm slices. Additionally, the lung tissue was perfused with 15% India ink from the trachea. The number of macroscopic metastatic nodules per lung was recorded to calculate the inhibitory effects on lung metastasis of breast cancer. Mouse blood was centrifuged at 4 ℃ for 10 min at 4,000 rpm, and serum was collected and stored at − 20 °C for analysis. The levels of blood AST, ALT, BUN, and Cr were detected using a blood biochemical analyzer [40].

The histopathology examination of tumor and lung sections was performed by H&E staining. Tissue sections were stained with primary antibodies against MMP-9, E-cad, CD31, Bcl-2, and Ki-67 (Abcam, Cambridge) to evaluate angiogenesis and cell proliferation in tumor and lung tissue. Quantification was performed blinded by counting positive cells in 10 fields (200 ×) using a light microscope (Zeiss, Germany).

Protein expression of p53, caspase 3, MMP-2, and vimentin in tumors was evaluated via western blot analysis. Tumor samples were collected and homogenized with the radioimmunoprecipitation assay lysis buffer. The extracted protein samples were separated by 4% ~ 12% sodium dodecyl sulfate–polyacrylamide gel electrophoresis and transferred onto polyvinylidene difluoride membranes. The membranes were incubated with the indicated primary antibodies including anti-p53 (1:1000), anti-caspase-3 (1:1000), anti-MMP-2, anti-vimentin (1:1000), and anti-GAPDH (1:2000) at 4 ℃ overnight. Membranes were incubated with a horseradish peroxidase-coupled secondary antibody, and signals were observed using the Pierce ECL Western Blotting Substrate (Thermo, Rockford, IL). The relative expression level of protein was quantified with ImageJ software.

### Statistical analysis

Data were expressed as the mean ± SD of at least three independent experiments. Data were analyzed using GraphPad Prism 8.0 (La Jolla, CA, USA). Statistical significance was determined by one-way analysis of variance, and a p-value of < 0.05 was considered significant.

## Supplementary Information

**Additional file 1.** Additional figures and tables.

## Data Availability

All data generated or analyzed during this study are included in this published article [and its supplementary information files].

## Abbreviations

TPL: Triptolide
HA-VE: Hyaluronic acid-Vitamin E succinate
PBAEss: Poly (β-Amino Esters)
GSH: Glutathione
TPL/NPs: TPL-loaded in HA-VE/PBAEss NPs
EPR: Enhanced permeability and retention
GPC: Gel permeation chromatography
DLS: Dynamic light scattering
TEM: Transmission electron microscopy
FT-IR: Fourier transform infrared spectroscopy
RSD: Relative standard deviation
XRD: X-ray diffraction
FITC: Fluorescein isothiocyanate
PI: Propidium iodide
FCM: Fow cytometry
CLSM: Confocal laser scanning microscopy
DiR: 1,10-Dioctadecyltetramethyl indotricarbocyanine iodide
NIR: Near-infrared
H&E: Hematoxylin and eosin
MMP-9: Matrix metalloproteinase-9
E-cad: E-cadherin
EMT: Epithelial-to-mesenchymal transition;
ALT: Alanine aminotransferase
AST: Aspirate aminotransferase
BUN: Urea nitrogen
Cr: Creatinine
