# Supplementary material for: Novel CD44-targeting and pH/redox-dual-stimuli-responsive core–shell nanoparticles loading triptolide combats breast cancer growth and lung metastasis
Source: J Nanobiotechnology. 2021 Jun 23;19:188. doi: 10.1186/s12951-021-00934-0 (PMC8220850; doi:10.1186/s12951-021-00934-0)
Supplement: Supplementary file 1 — Additional file 1. Additional figures and tables. [file 12951_2021_934_MOESM1_ESM.docx]

**Supporting Information**

**Novel CD44-targeting and pH/redox dual-stimuli responsive core-shell nanoparticles loading triptolide to combat breast cancer growth and lung metastasis**

Jinfeng Shi**^†^**, Yali Ren**^†^**, Jiaqi Ma, Xi Luo, Jiaxin Li, Yihan Wu, Huan Gu, Chaomei Fu, Zhixin Cao*, Jinming Zhang*

State Key Laboratory of Southwestern Chinese Medicine Resources, Pharmacy School, Chengdu University of Traditional Chinese Medicine, Chengdu, China

† These authors have contributed equally to this work and share the first authorship.

*** Correspondence:**

Dr. Zhixing Cao, No.1166 Liutai Avenue,Wenjiang District, Chengdu city, College of Pharmacy, Chengdu University of Traditional Chinese Medicine, Chengdu, China

Tel.: +86 15882236641

E-mail address: [caozhixing@cdutcm.edu.cn](mailto:caozhixing@cdutcm.edu.cn)

Dr. Jinming Zhang, No.1166 Liutai Avenue,Wenjiang District, Chengdu city, College of Pharmacy, Chengdu University of Traditional Chinese Medicine, Chengdu, China

Tel.: +86 13551043885

E-mail address: [cdutcmzjm@126.com](mailto:cdutcmzjm@126.com)

**Methods**

**Drug loading efficiency and drug entrapment efficiency of TPL/NPs**

To determine the drug loading efficiency (DLE) and drug entrapment efficiency (DEE) in TPL/NPs, 1 mg of lyophilized TPL/NPs was dissolved in 4 mL of methanol with ultrasonication for 2 min. The concentration of TPL was tested by high performance liquid chromatography (HPLC) (LC-45202-46, SHIMADZU, Japan) equipped with a C18 column (250×4.6 mm) and a UV detector. The mobile phase was methanol/water (v/v, 46%: 54%) with a flow rate of 1 mL/min and UV absorbance was detected at a wavelength of 220 nm at 25 °C. The DLE and DEE were calculated as following formulas (1) and (2)(Luo et al., 2020):

DLE (%) = $\frac{The amount of drug in nanoparticles}{The amount of added polymer and drug}$×100% (1)

DEE (%) = $\frac{The amount of drug in nanoparticles}{The amount of added drug}$×100% (2)

**Characterization of TPL/NPs**

The size and zeta-potential of TPL/NPs were determined by dynamic light scattering (DLS) with a Malvern Zetasizer (Nano ZS90, Malvern Instruments, UK) at room temperature. The morphology of TPL/NPs was observed by Transmission Electron Microscopy (TEM, JEM 1200X, JEOL, Japan) with an accelerating voltage of 200 kV. A suspension of TPL/NPs was placed on a copper grid, negatively stained with phosphotungstic acid for 30 s and dried at room temperature. Additionally, the thermal analysis of TPL/NPs was analyzed using X-ray diffraction (XRD). XRD analysis was performed on a diffractometer (D8 Advance, BRUKER, Germany) by scanning from 5° to 90° at a speed of 6° per min and operating at 40 kV and 40 mA. The infrared (IR) spectra of TPL/NPs were recorded using a FT-IR spectrophotometer (IRTracer-100, Shimadzu, Japan). Briefly, samples were blended with KBr, and the resulting mixtures were further pressed in a pancake shape prior to measuring.

**Stability experiment**

The stability of TPL/NPs was determined by measuring particle size (nm) and polydispersity index (PDI) using a Zetasizer Nano ZS 90 during the storage time (0, 1, 2, 3, 4, 5, 6, 7 and 14 d) at 2~8°C. Additionally, the mean particle sizes of TPL/NPs in PBS (pH 7.4) with or without 10% FBS at 4 °C was also measured by DLS at specific time points (0, 2, 12, 24, 48, 72 and 96 h).

**In vitro drug release study**

The release of TPL from NPs was determined using dialysis (MWCO 3000). It was studied under shaking (100 rpm) at 37 °C in four different media which were the phosphate buffer saline (PBS, pH 7.4) containing 1% Tween 80, PBS (pH 7.4) containing 1% Tween 80 with 10 mM GSH, phosphate buffer saline (pH 5.8) containing 1% Tween 80, phosphate buffer saline (pH 5.8) containing 1% Tween 80 with 10 mM GSH, respectively. Typically, 2 mL of TPL-loaded NPs dispersions was added to 30 mL of release media. The release studies were conducted at different time intervals: 0.5, 1, 2, 4, 6, 8, 10, 12, and 24 h. At certain times, 1 mL outside buffer solution was taken out and the media was refreshed. The TPL concentration in the release medium was determined by HPLC. Each experiment was repeated in triplicate and the results were expressed by mean ± SD.

**In vitro hemolysis**

Rabbit blood samples were collected from heart blood into test tubes containing heparin sodium, centrifuged and washed with saline. 0.2 mL of the obtained RBCs was diluted with saline to 10 mL. 0.5 mL of the RBCs suspension was incubated with 0.5 mL of the NPs suspensions (1, 10, 20, 30 and 50 μg/ml) at 37°C with gentle shaking. After 4 h, the samples were centrifuged at 3000 rpm for 10 min. The absorbance (A) of the supernatant was measured by micro plate spectrophotometer at 545 nm. A negative control was prepared by mixing 0.5 mL of the RBC suspension with 0.5 mL of saline (0% lysis), using water as a positive control (100% lysis). The hemolytic rates of the samples were calculated using the following equation:

Hemolytic rate (%) = $\frac{A_{t}-A_{nc}}{A_{pc}-A_{nc}}$×100%

Where A_t_ represents absorbance value of test sample, A_nc_ and A_pc_ stand for absorption value of negative and positive controls, respectively.

**Immunofluorescence assay of CD44 expression in cell lines**

**The CD44 expression was quantified by FCM.** MDA-MB-231, MCF-7 and PC 12 cells were seeded into the six-well plate at a density of 2×10^5^ cells/well and incubated for 24 h at 37°C, 5% CO_2_ and 10% humidity. When the cells grew to the logarithmic stage, they were digested with trypsin, collected, centrifuged at 800 rpm for 5 min, washed twice with PBS, and re-suspended with PBS to adjust the cell concentration to be 1×10^6^/100 μL PBS. Add 10μl labeled antibody to the bottom of flow tube mixing with the cells, incubate for 20 min at 4°C away from light. After incubation, add 2 ml PBS washing twice. Add 0.5 ml PBS to re-suspend the cells and detect by flow cytometry.

**The CD44 expression in MDA-MB-231, MCF-7 and PC 12 cells was also determined by confocal laser scanning microscopy.** The cells were seeded into 35 mm dishes for 24 h. Add 10 μL labeled antibody to the dishes mixing with the cells, incubate for 20 min at 4 °C away from light. After incubation, add 2 mL PBS washing twice and fixed in ice cold 4% paraformaldehyde for 10 min. The cell nuclei were stained with Hoechst 33342 for 10 min and then monitored by Olympus IX51 Fluorescence microscopy.

***In vivo* biodistribution of TPL/NPs and free TPL**. Biodistribution study was conducted in breast tumor-bearing BALB/c mice (subcutaneously injected with 1×10^6^ 4T1 cells). Mice were injected with TPL/NPs, or free TPL of the equivalent TPL dose (1 mg/kg, n=3) via the tail vein. At selected time points (2, 6, 12, and 24 hours) post-injection, tumor and liver were excised from euthanized animals. Three volumes of saline were added into tissues to obtain homogenates. And then, two volumes of acetonitrile were added to tissue homogenates. The supernate was concentrated by N_2_ and measured via LC-MS (Q Exactive Orbitrap, Thermofisher Scientific). Quantification of TPL was performed using LC-MS with positive electrospray ionization in multiple reaction monitoring mode with an ion pair (m/z 361.3→128.2)^[2]^.

**Results**

**Table S1** Physical and chemical properties of different preparation of TPL/NPs.

| Samples | HA-VE (mg) | PBAEss (mg) | Particle size (nm) | DLE (%) | DEE (%) |
| --- | --- | --- | --- | --- | --- |
| 1^#^ | 0 | 10 | 152.58 ± 4.37 | 9.38 ± 0.71 | 56.29 ± 4.1 |
| 2^#^ | 5 | 10 | 172.33 ± 5.42 | 8.75 ± 0.54 | 74.34 ± 3.8 |
| 3^#^ | 10 | 10 | 191.31 ± 7.08 | 8.63 ± 0.88 | 94.93 ± 2.1 |
| 4^#^ | 15 | 10 | 223.14 ± 19.66 | 7.06 ± 0.67 | 95.31 ± 3.3 |
| 5^#^ | 20 | 10 | 268.95 ± 13.60 | 6.02 ± 0.55 | 96.27 ± 2.2 |

**Table S2** Characterization of PBAEss recorded by GPC.

| Polymer | Yield (%) | Mn (1×10^4^) | Mw (1×10^4^) | PDI |
| --- | --- | --- | --- | --- |
| PBAEss | 72.3 | 1.75 | 3.51 | 2.00 |


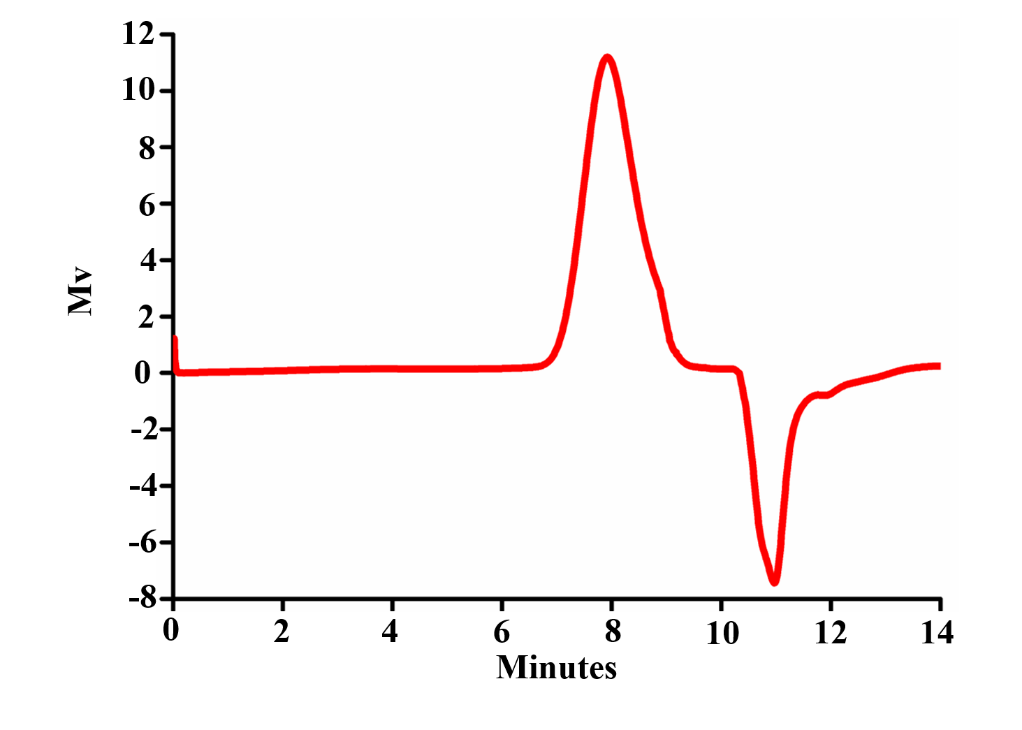


**Figure S1** GPC chromatogram of PBAEss.


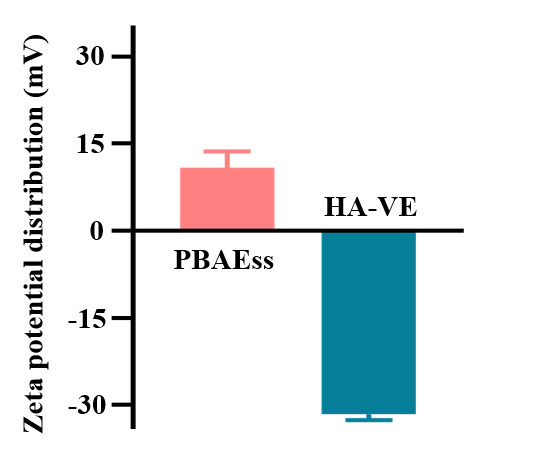


**Figure S2** Zeta potential measurement of PBAEss and HA-VE solution.


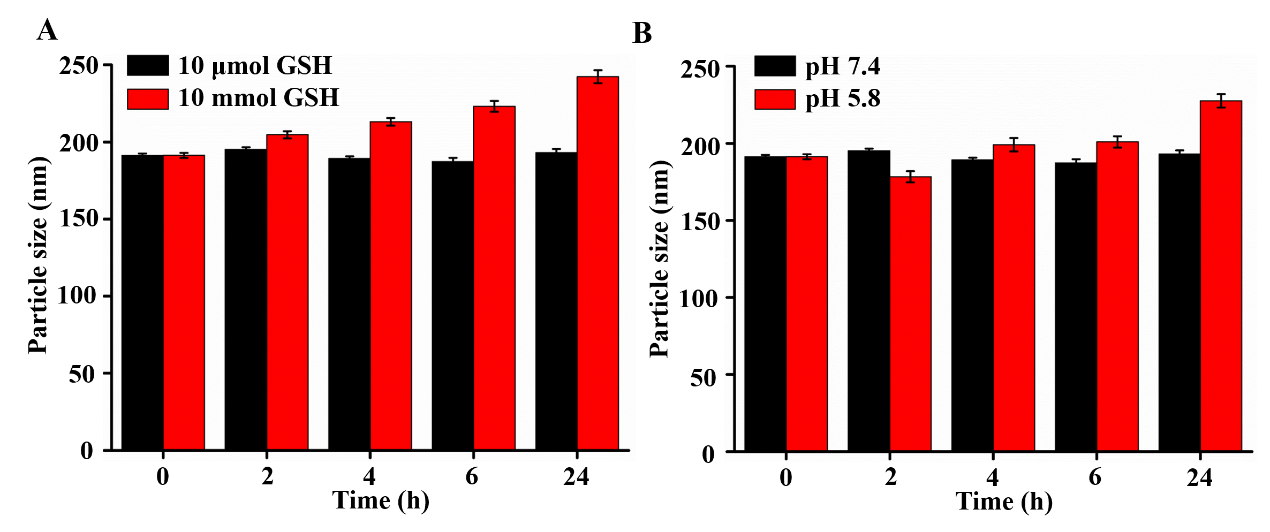


**Figure S3** (A) Particle size of TPL/NPs in response to redox-stimulus monitored by DLS. (B) Particle size of TPL/NPs in response to pH-stimulus monitored by DLS.


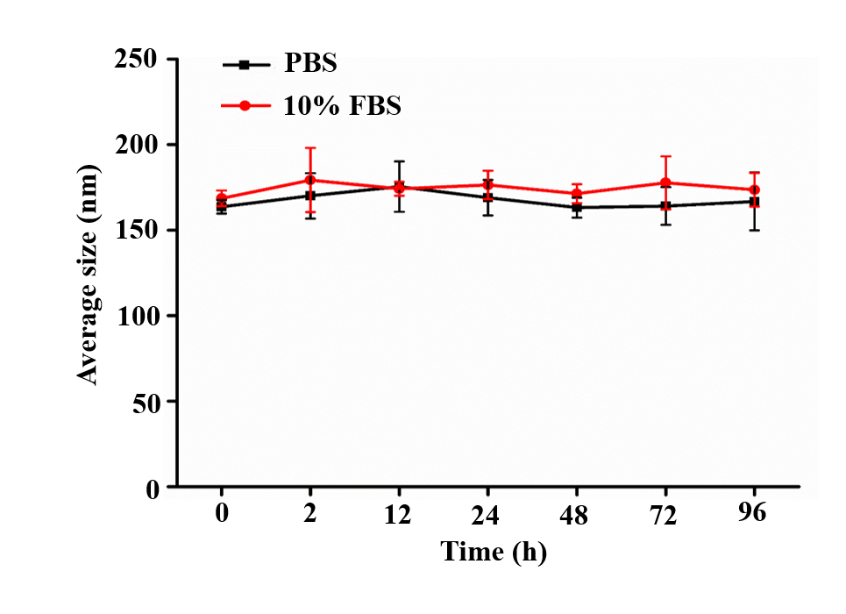


**Figure S4** Stability of TPL/NPs in PBS and 10% FBS.


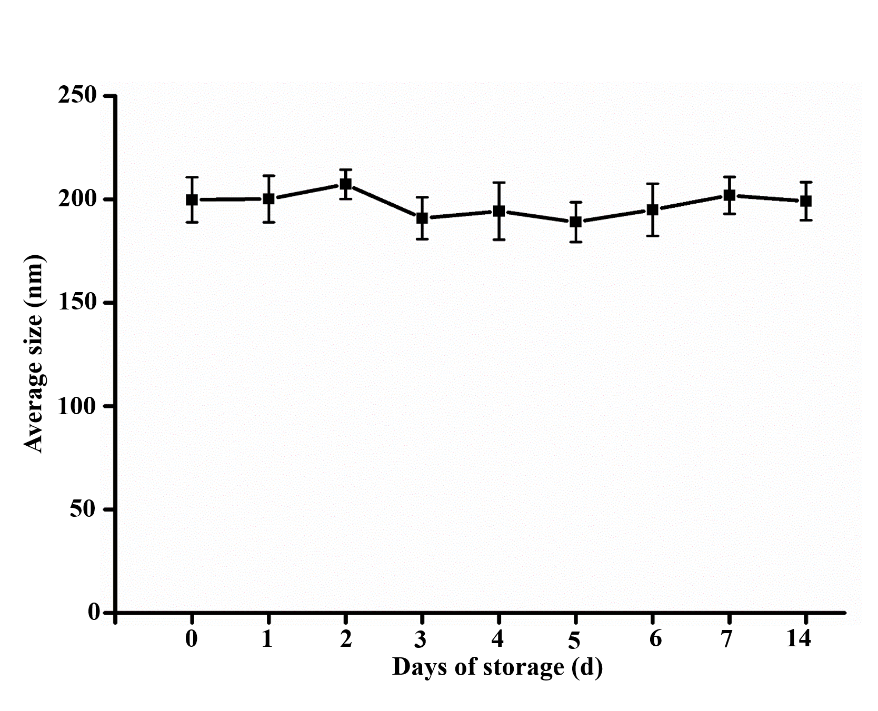


**Figure S5** Stability of TPL/NPs.


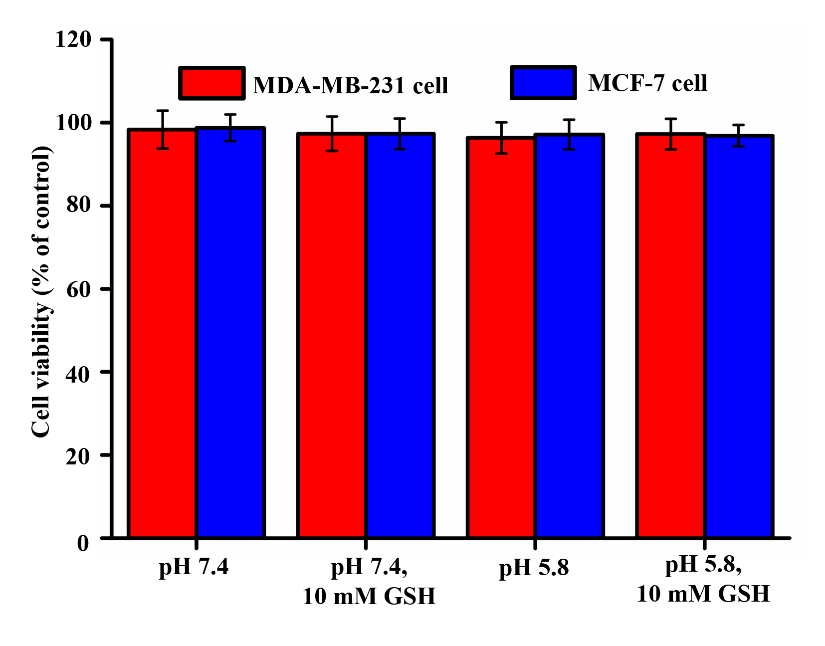


**Figure S6** Cytotoxicity of Blank NPs against MDA-MB-231 and MCF-7 cells were treated with various medium for 48 h, respectively.


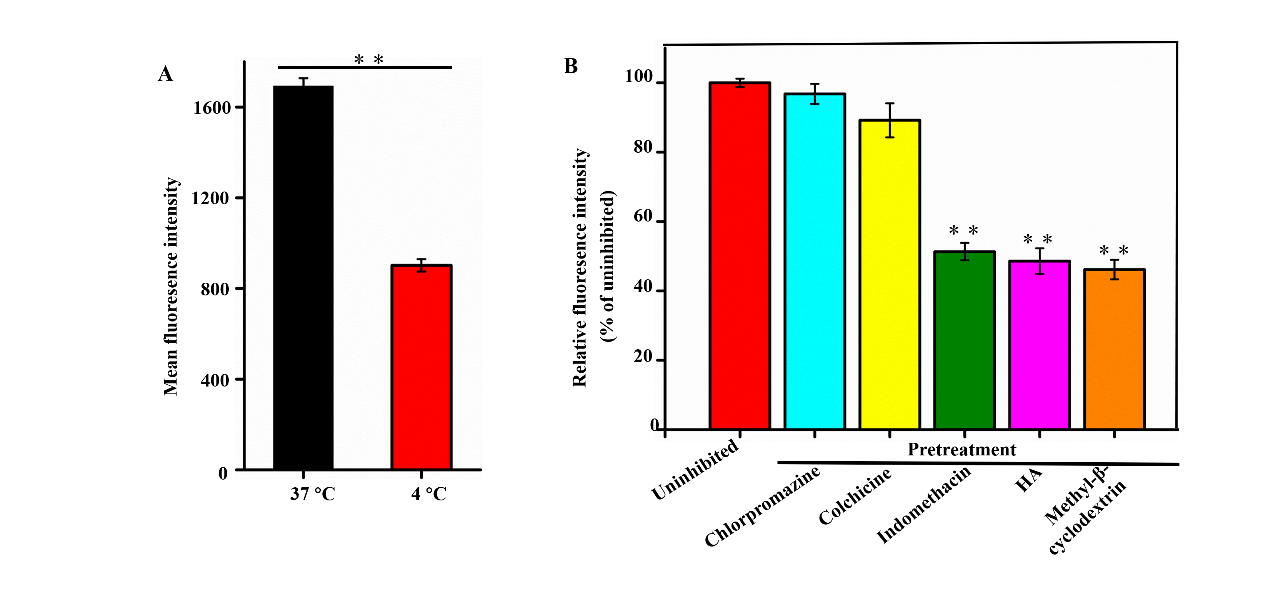


**Figure S7** (A) Relative fluorescence intensity of C6/NPs after incubated at 4˚C and 37˚C. (B) Relative fluorescence intensity of C6/NPs for 4 h incubation in MDA-MB-231 cells with or without pretreated by endocytosis inhibitors. Indicated values were mean ± SD (n=3), *p<0.05, **p<0.01, significant difference between free C6 and C6/NPs groups.


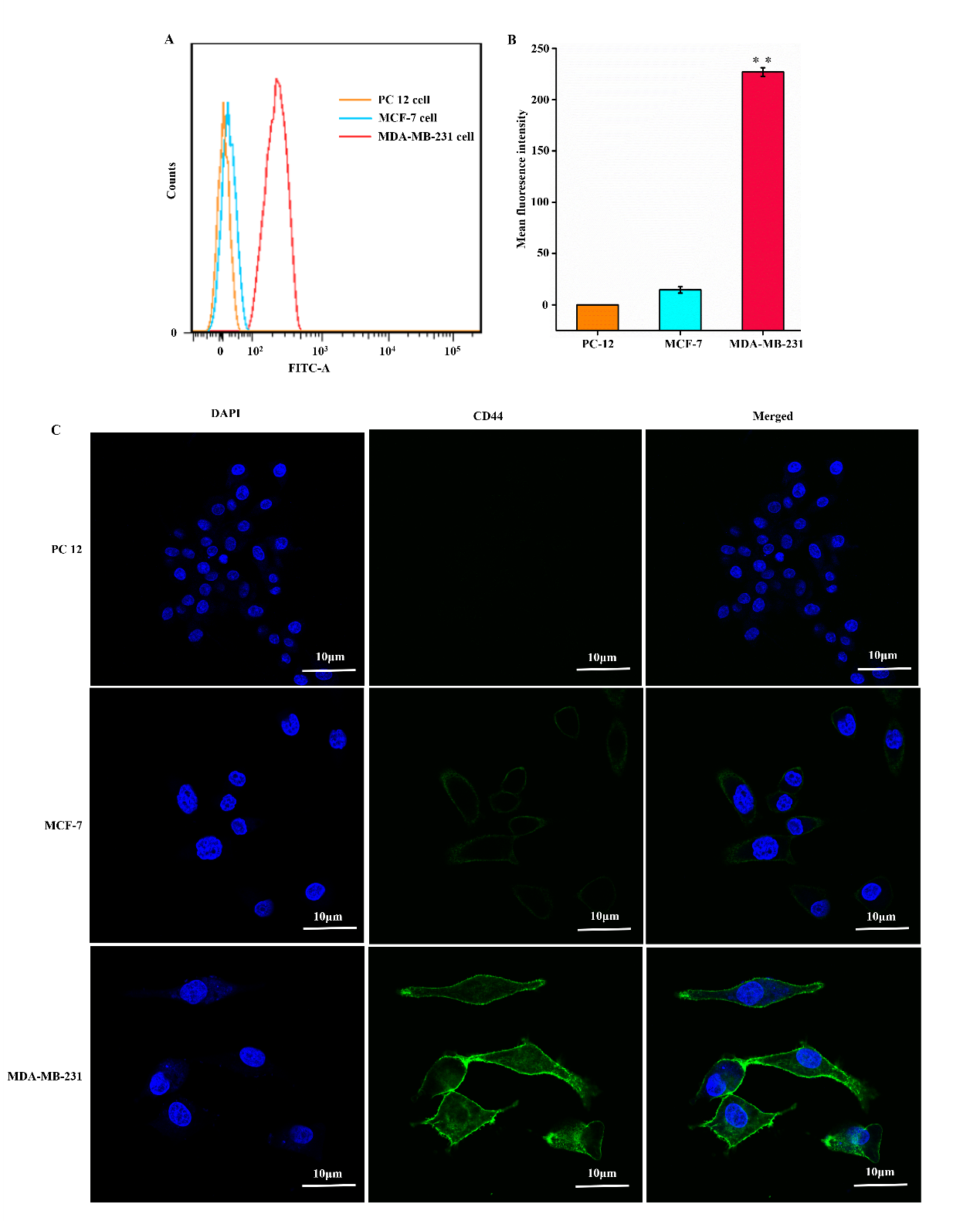


**Figure S8** (A) Flow cytometric curves of immunofluorescent detection of CD44 expression on MDA-MB-231 and MCF-7 cell lines. (B) Flow cytometry measurement of the immunofluorescent detection of CD44 expression on MDA-MB-231 and MCF-7 cell lines. **p<0.01 versus MCF-7. (C) Immunofluorescent detection of CD44 expression on MDA-MB-231 and MCF-7 cell lines. PC 12 cells were selected as the control. Blue: nuclei stained by Hoechst 33342; and green: CD44 stained by FITC labeled antibody.


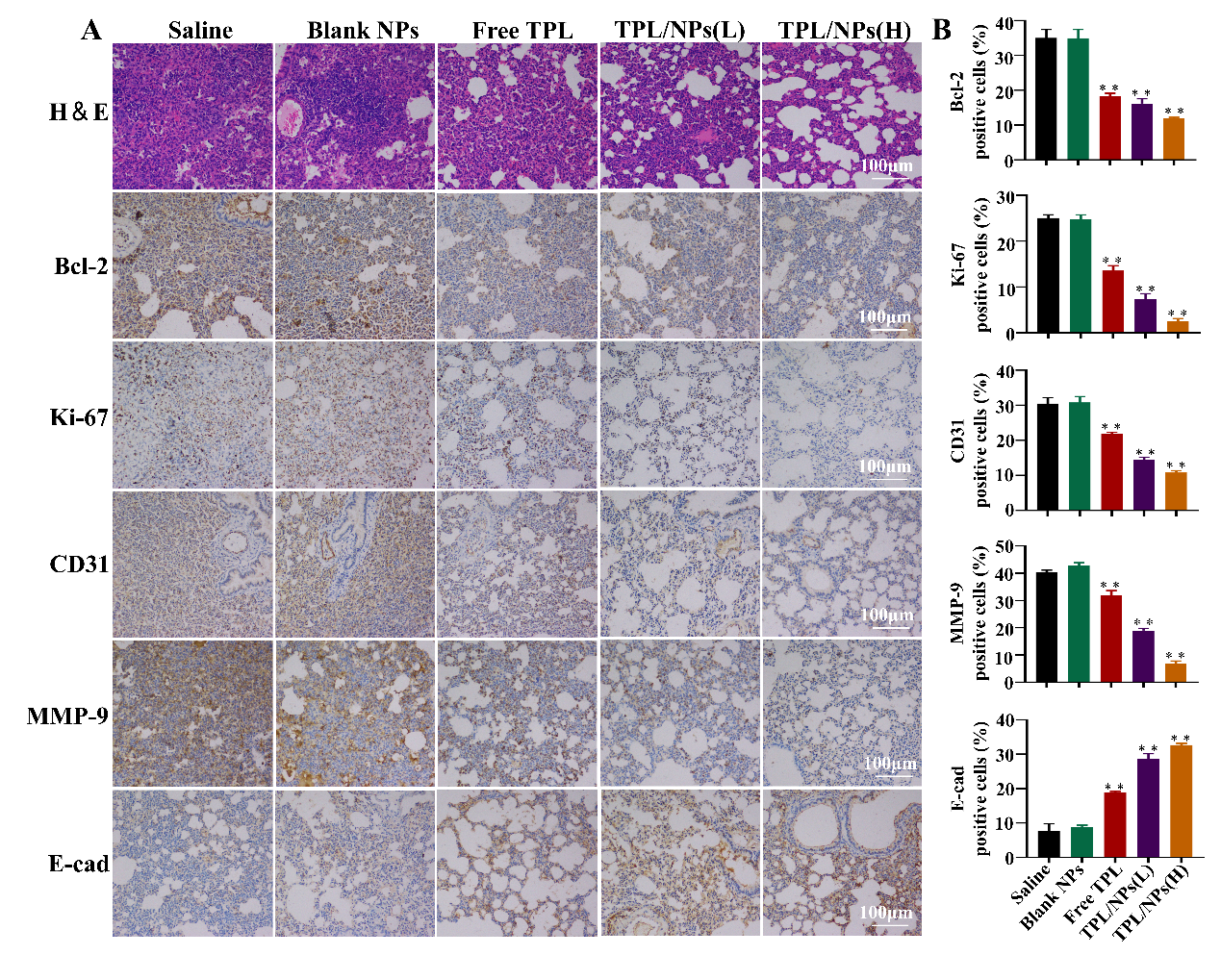


**Figure S9** Histological staining and Immunohistochemistry staining of representative proteins in lung sections derived from mice treated with saline, free TPL, Blank NPs, TPL/NPs(L) and TPL/NPs(H), respectively. (A) H&E staining and immunohistochemistry staining of Bcl-2, Ki-67, CD31, MMP-9 and E-cad; (B) Statistical analysis for the percentage of Bcl-2, Ki-67, CD31, MMP-9 and E-cad expression in the tumor sections by immunohistochemistry staining. Note: *p<0.05.

**Reference:**

[1]Luo, R. F., Lin, M. S., Zhang, C., Shi, J. F., Zhang, S. Y., Chen, Q. Y., et al. (2020). Genipin-crosslinked human serum albumin coating using a tannic acid layer for enhanced oral administration of curcumin in the treatment of ulcerative colitis. *Food Chemistry* 330, 10. doi: 10.1016/j.foodchem.2020.127241.

[2] Ye Xu, Xiaoyan Chen, Dafang Zhong (2019). A sensitive LC-MS/MS method for the determination of triptolide and its application to pharmacokinetic research in rats. *Biomed Chromatogr*. 2019,33(3):e4422
